# Two FERONIA-Like Receptor Kinases Regulate Apple Fruit Ripening by Modulating Ethylene Production

**DOI:** 10.3389/fpls.2017.01406

**Published:** 2017-08-10

**Authors:** Meiru Jia, Ping Du, Ning Ding, Qing Zhang, Sinian Xing, Lingzhi Wei, Yaoyao Zhao, Wenwen Mao, Jizheng Li, Bingbing Li, Wensuo Jia

**Affiliations:** College of Horticulture, China Agricultural University Beijing, China

**Keywords:** apple, ethylene production, fruit ripening, FERONIA-like receptor kinase, tomato, VIGS

## Abstract

Ethylene has long been known to be a critical signal controlling the ripening of climacteric fruits; however, the signaling mechanism underlying ethylene production during fruit development is unknown. Here, we report that two FERONIA-like receptor kinases (FERLs) regulate fruit ripening by modulating ethylene production in the climacteric fruit, apple (*Malus*×*domestica*). Bioinformatic analysis indicated that the apple genome contains 14 members of the FER family (*MdFERL1–17*), of these 17 FERLs, *MdFERL6* was expressed at the highest level in fruit. Heterologous expression of *MdFERL6* or *MdFERL1*, the apple homolog of Arabidopsis *FER*, in another climacteric fruit, tomato (*Solanum lycopersicum*) fruit delayed ripening and suppressed ethylene production. Overexpression and antisense expression of *MdFERL6* in apple fruit calli inhibited and promoted ethylene production, respectively. Additionally, virus-induced gene silencing (VIGS) of *SlFERL1*, the tomato homolog of *FER*, promoted tomato fruit ripening and ethylene production. Both MdFERL6 and MdFERL1 physically interacted with MdSAMS (S-adenosylmethionine synthase), a key enzyme in the ethylene biosynthesis pathway. *MdFERL6* was expressed at high levels during early fruit development, but dramatically declined when fruit ripening commenced, implying that MdFERL6 might limit ethylene production prior to fruit development and the ethylene production burst during fruit ripening. These results indicate that FERLs regulate apple and tomato fruit ripening, shedding light on the molecular mechanisms underlying ripening in climacteric fruit.

## Introduction

Fleshy fruits are physiologically classified as climacteric or non-climacteric. Climacteric fruits exhibit an increase in respiration at the onset of ripening (Nitsch, 1953; Coombe, 1976; Brady, 1987). Studies of the mechanisms regulating fruit ripening began in the 1920s (Brady, 1987) and a major focus has been identifying the critical internal factors or signals governing this process. Ethylene has long been known to be a critical signal controlling the ripening of climacteric fruit (Biale, 1964; Burg and Burg, 1965; Alexander and Grierson, 2002), which exhibit a large increase in ethylene production at the onset of ripening. Exposure to exogenous ethylene can initiate the ripening of climacteric fruits (Seymour et al., 2013), and its effect is so great that limiting ethylene production in fruits or ethylene exposure for harvested fruit has become a major concern in the commercial cultivation industry (Brady, 1987).

The ripening of fleshy fruits is a complex process that involves dramatic changes in physiological and biochemical metabolism (Giovannoni, 2001; Seymour et al., 2013). While early studies focused on these changing patterns, more recent research has aimed to establish the molecular basis for the regulation of fruit development and ripening (Seymour et al., 2013). Several genes associated with the regulation of fruit development and ripening have been identified, but as most encode enzymes or transcription factors, little is known about the signaling mechanisms that underlie these processes to control the changes in physiological and biochemical metabolism (Fischer and Bennett, 1991; Giovannoni, 2001; Klee and Giovannoni, 2011; Seymour et al., 2013).

Most studies of fruit development-associated signaling have focused on the molecular perception of and response to ethylene in the model plant, tomato (*Solanum lycopersicum*; Felix et al., 1991; Wang et al., 2002; Bram et al., 2015). The mechanisms controlling the increase in ethylene production are essential for its function as a signal regulating fruit development and ripening. Ethylene production is primarily controlled by the activities of the key enzymes in its biosynthesis pathway. In the context of cellular signaling, enzyme activity may be regulated at the transcriptional or post-transcriptional level by upstream signaling cascades. While ethylene biosynthesis and its downstream signaling pathways have been studied extensively, little is known about the signaling cascades upstream of ethylene biosynthesis. However, it was shown that ethylene biosynthesis is induced by both cell wall fragments and wall-lysing enzymes. Wall fragments were implicated as possible regulators of ethylene biosynthesis in ripening citrus fruit (*Citrus* sp.; Baldwin and Biggs, 1988), tomato fruit (Baldwin and Pressey, 1988; Brecht and Huber, 1988), and cultured pear (*Pyrus* sp.) cells (Tong et al., 1986), and the wall-lysing enzymes polygalacturonase and endoxylanase were reported to induce ethylene biosynthesis in tomato fruit (Baldwin and Pressey, 1988) and tobacco (*Nicotiana tabacum*) leaves (Fuchs et al., 1989), respectively. Although the precise mechanism by which cell wall degradation induces ethylene biosynthesis is not known, these related studies suggest that receptors or sensors that initiate the signaling cascade upstream of ethylene biosynthesis exist in the membrane–wall complex.

Plant receptor-like kinases (RLKs) are transmembrane proteins with putative amino-terminal extracellular domains and carboxyl-terminal intracellular kinase domains (Shiu and Bleecker, 2001a; Humphrey et al., 2007; Boisson-Dernier et al., 2011; Cheung and Wu, 2011; Lindner et al., 2012). The *Arabidopsis thaliana* genome contains more than 600 RLK homologs with diverse functions (Shiu and Bleecker, 2001a,b, 2003). We hypothesize that RLKs function as membrane–wall complex-anchored receptors that regulate ethylene biosynthesis during fruit development and ripening. The RLK superfamily contains a subfamily with extracellular malectin or malectin-like domains that putatively bind oligosaccharides (Verica and He, 2002; Annabelle et al., 2006; Alexandre et al., 2010; Schallus et al., 2010). Oligosaccharides have increasingly been demonstrated to function as signals that activate either defensive or developmental processes in plants (Clarence and Farmer, 1991; Schallus et al., 2008), and therefore elucidating whether malectin or maletin domain-containing RLKs are implicated in the regulation of fruit development and ripening is a key research target. In a previous study, we found that the strawberry genome (*Fragaria vesca*) contains 62 RLK members (designated FvMRLKs) that contain malectin or malectin-like domains, and a preliminary analysis suggested that more than half of the FvMRLKs could be implicated in strawberry fruit development and ripening (Zhang et al., 2016).

Recently, several malectin domain-containing RLKs have been demonstrated to play important roles in Arabidopsis growth and development (Nissen et al., 2016); among these genes, *FERONIA* (*FER*) is of particular interest because of its diverse roles in the regulation of a series of crucial biological processes. FER was first identified for its role in fertilization (Huck et al., 2003). In Arabidopsis, FER directly interacts with Rho of plants guanine nucleotide exchange factors (RopGEFs), activating the ROP GTPase and mediating the production of reactive oxygen species (ROS) at the entrance of the female gametophyte, thereby inducing pollen tube rupture and sperm release (Kessler et al., 2010; Duan et al., 2014; Ngo et al., 2014). FER is believed to function in a variety of other important processes, such as root hair elongation (Duan et al., 2010; Huang et al., 2013), starch accumulation (Yang et al., 2015), sugar utilization (Pu et al., 2017), seed development (Yu et al., 2014), pathogen resistance (Keinath et al., 2010; Kessler et al., 2010), and vegetative growth (Guo et al., 2009; Deslauriers and Larsen, 2010). Notably, FER has been demonstrated to play a pivotal role in the cross-talk signaling among the phytohormones that play crucial roles in *Arabidopsis*, including abscisic acid (ABA; Yu et al., 2012; Chen et al., 2016), auxin (Duan et al., 2010), brassinosteroids (BR), and ethylene (Guo et al., 2009; Deslauriers and Larsen, 2010). Importantly, a recent study by Mao et al. (2015) reported that FER was able to physically interact with S-adenosylmethionine synthetase (SAMS) and thereby suppress ethylene biosynthesis in *Arabidopsis*.

Given the involvement of FER in a variety of important processes, especially in the cross-talk between phytohormones and in ethylene biosynthesis in *Arabidopsis*, we were particularly interested to determining whether some FER-like RLKs regulate fruit development and ripening. Apple (*Malus* × *domestica*) is one of the most popular and important fruit crops worldwide; however, due to difficulties in molecular research using apple fruit, basic research into fruit development and ripening has commonly been conducted using tomato as a model plant. In the present study, we searched the apple genome database and identified 14 FER-like RLKs. We demonstrated that two of these proteins are important regulators of fruit development and ripening. Interestingly, two of the FER-like RLKs were found to be regulators of ethylene production in fruits. This work sheds light on the mechanisms underlying climacteric fruit development and ripening, by suggesting that Feronia-like RLKs represent upstream signaling components that regulate ethylene production during ripening.

## Materials and methods

### Plant materials and growth conditions

Tomatoes (*Solanum lycopersicum* L. cv. Micro Tom) were planted in pots (diameter, 20 cm; depth, 20 cm) containing a mixture of nutrient soil, vermiculite, and organic fertilizer (4:2:1, v/v/v). Tomato seedlings were grown under standard conditions: 60% relative humidity and 25°C/18°C (day/night) under a 12 h/12 h light/dark cycle. Plants were watered daily to the drip point. Apple (*Malus* × *domestica*) callus tissue derived from the “Golden Delicious” cultivar was grown on Murashige and Skoog (MS) medium at 27°C ± 1°C in darkness, and subcultured at 10-day intervals before being used for gene transformation.

### Gene isolation and sequence analysis

The cDNA sequences of full-length Arabidopsis CrRLK1Ls were obtained from TAIR (http://www.arabidopsis.org). To identify *FER-like* (*FERL*) genes in apple or rice, the coding sequence of *FER* (At3g51550) was used as a query to BLAST the apple genome database (http://genomics.research.iasma.it/) or NCBI (https://www.ncbi.nlm.nih.gov/). Phylogenetic trees were constructed using the Neighbor-Joining (NJ) method in MEGA 4.0.2 software (Tamura et al., 2007), with 1000 bootstrap replicates performed to evaluate the reliability of the different phylogenetic groups. The deduced amino acid sequences of the MdFERLs were aligned using ClustalX 2.0.12 (Larkin et al., 2007) with default settings. The alignments were edited and marked using GeneDoc (Nicholas et al., 1997).

To isolate *MdFERL1* and *MdFERL6*, total RNA was extracted from the fruit using an EZNATotal RNA Kit (Omega Biotek), according to the manufacturer's instructions. First-strand cDNA synthesis was conducted from 1 μg of total RNA. Full-length *MdFERL1* and *MdFERL6* were cloned from cDNA by PCR using Q5 High-Fidelity DNA Polymerase (New England Biolabs) and the following conditions: 98°C for 30 s (one cycle); 98°C for 30 s, 59°C for 25 s, and 72°C for 5 min (35 cycles); with a final extension of 72°C for 2 min. The PCR products were subcloned into the pCambia1304 vector and used to produce positive colonies. The primer sequences and GenBank accession numbers are shown in Supplemental Table S1.

### Quantitative reverse transcription PCR (RT-qPCR)

RT-qPCR was performed using SYBR Premix Ex TaqTM (Takara Bio) in a 7500 Real-Time PCR System (Applied Biosystems). The expression level of each gene was analyzed in three pools of five fruits, with three measurement replicates per pool. *ACTIN* was used as an internal control. The 2^−ΔΔCT^ method was used to determine transcription levels, where ΔCT represents the difference between the cycle threshold values of the target and reference genes (Schmittgen and Livak, 2008).

### Spatio-temporal expression of *MdFERL*s and ripening marker genes

The whole process from fruit set to ripening was classified into five stages based on days post-anthesis (DPA), namely 60DPA, 85DPA, 105DPA, 130DPA, and 155DPA. The fruit was frozen in liquid nitrogen, and kept at –80°C until required for gene expression analysis. The expression of *MdFERL* and ripening marker genes was assessed by RT-qPCR analysis, using primers designed in Primer3 Plus (http://www.primer3plus.com/cgi-bin/dev/primer3plus.cgi). Primer sequences are shown in Supplemental Tables S2, S3, S5. The data from five fruits were combined as an individual sample and each sample was analyzed in triplicate.

### Analysis of *MdFERL* expression changes in response to 1-MCP and ACC treatment

For the apple fruit treatment, 5 g of fruit disks (10 mm in diameter and 1 mm in thickness) was prepared and combined from six fruits at the 105 DPA stage. The disk samples were vacuum infiltrated for 30 min in equilibration buffer (10 mM MgCl_2_, 50 mM MES-Tris (pH 5.5), 5 mM CaCl_2_, 10 mM EDTA, 200 mM mannitol, and 5 mM vitamin C) (Archbold, 1988). The samples were then shaken for 6 h at 25°C in equilibration buffer containing 1 mM ACC or 3 μM 1-MCP. After incubation in the dark for 6 h, the samples were washed and frozen immediately in liquid nitrogen, and maintained at –80°C until required. Each individual analysis was conducted with three sample replicates. Primers used for the RT-qPCR analysis of ripening-related genes are provided in Supplemental Table S2.

To treat apple calli with 1-MCP, the calli were transfected with *MdFERL6*-AS and empty pCambia1304 vector, and then cultivated on MS medium at 27°C in darkness. Three days after the transfection, calli were treated with 3 μM 1-MCP for 6 h and expression of the selected genes was analyzed as described above.

### Transfection of tomato fruit and apple callus by agroinfiltration

To construct vectors for the overexpression (OE) of *MdFERL1* (MDP0000445374) and *MdFERL6* (MDP0000465341) (abbreviated hereafter as *MdFERL1-OE* and *MdFERL6-OE*), full-length *MdFERL1* and *MdFERL6* were respectively cloned into the plant expression vector pCambia1304 using the *Kpn*I and *Eco*RI restriction sites. The empty pCambia1304 vector and the overexpression vectors *MdFERL1-OE* and *MdFERL6-OE* were transformed individually into the *Agrobacterium tumefaciens* strain EHA105 (Lazo et al., 1991). For the *MdFERL6*-antisense (as) vector, a 621-bp fragment near the 5′ end of *MdFERL6* cDNA was amplified by PCR using the primer pair 5′-GAATTCGTCCACGAACGTGTCTC -3′ (with an *Eco*RI restriction site; forward) and 5′- GGTACCGGATCGTTGATCTCAGAG -3′ (with a *Kpn*I restriction site; reverse). The obtained fragment was sequenced and forward-cloned into the *Eco*RI and *Kpn*I restriction sites of pCambia1304. To generate the TRV-VIGS (virus-induced gene silencing) vectors, a 595-bp fragment of *SlFERL1* (GenBank accession number KY435602) was PCR-amplified from tomato cDNA. The resulting product was cloned into the vector pTRV2 to generate pTRV2-*SlFERL1*. The *A. tumefaciens* strain GV3101 containing vectors pTRV1 or pTRV2 and their derivatives was used for the VIGS experiments.

The transformed strains were grown at 28°C in Luria-Bertani liquid medium containing 10 mM MES, 20 μM acetosyringone, and the appropriate antibiotics. When the culture reached an optical density at 600 nm of approximately 0.8, the *A. tumefaciens* cells were harvested, resuspended in infection buffer (10 mM MgCl_2_, 10 mM MES (pH 5.6), and 200 mM acetosyringone), and shaken for 2 h at room temperature before being used for infiltration. For VIGS infiltration, each *A. tumefaciens* strain containing pTRV1 and pTRV2 or their derivative vectors was mixed at a ratio of 1:1 and infiltrated into the carpopodium of tomato fruits attached to the plant about 18 days after pollination, using a 1-mL needle-less syringe. Pairs of fruit at the same developmental stage and with similar phenotypes were selected; for each pair, one fruit was transfected with *MdFERL1*-OE, *MdFERL6*-OE, or *SlFERL1*-VIGS, while the other was transfected with an empty vector as the control. For each gene, 25 pairs of fruit were injected. To examine the effect of *MdFERL1*-OE, *MdFERL6*-OE, and *SlFERL1*-VIGS on the expression of ripening-related genes, the fruits were collected 10 dayd after transfection for *MdFERL1*-OE and *MdFERL6*-OE, and 12 days after transfection for *SlFERL1*-VIGS.

Callus tissues were subcultured three times at 15-day intervals before being used for gene transformation. The fresh calli were soaked for 20 min in an *A. tumefaciens* solution containing the *MdFERL6*-OE or *MdFERL6*-AS constructs, or the empty vector. The calluses were co-cultured on MS medium at 25°C.

### Determination of fruit ripening-associated physiological parameters

Flesh firmness was measured after skin had been removed on opposite sides of the fruit using a GY-4 fruit hardness tester (Zhejiang Top Instrument). The contents of fruit pigment, flavonoid, and total phenol were determined as described (Fuleki and Francis, 1968; Lees and Francis, 1971). The soluble sugar content was determined as described by Jia et al. (2011). Titratable acidity was calculated as malic acid. The total titratable acidity was determined using the acid-base titration method (Kafkas et al., 2007). Fruit aroma production was characterized by performing a headspace solid-phase microextraction and gas chromatography-mass spectrometry, as described by Dong et al. (2013).

### Measurements of ethylene production

To measure ethylene production, intact fruits were enclosed in a gas-tight container (0.86 L, 24°C) equipped with septa, and 1 mL of headspace gas was sampled using a gas syringe. The ethylene concentration was measured using a gas chromatograph (GC-17A, Shimadzu). Six fruits were assessed as one sample, and three replicates were performed.

### Bimolecular fluorescence complementation (BiFC) and subcellular localization assays

For BiFC assays, the full-length coding sequences of *MdFERL1, MdFERL6, MdSAMS, MdETR2, MdETR5, MdACS3*, and *MdACS5* were amplified and individually cloned into pCambia1300-YFP(n/c) vectors. To determine the subcellular localization of *MdFERL6*, the full-length cDNA sequence was amplified and fused to green fluorescent protein (GFP) in the pMDC83 plant expression vector. A coexpression analysis was conducted in tobacco (*Nicotiana tabacum*) leaves as described by Schütze et al. (2009). Fluorescence was observed 3 days after transformation using a confocal laser-scanning microscope (Fluoview FV1000, Olympus). Maize (*Zea mays*) protoplast isolation and transformation were carried out according to a protocol described by Sheen et al. (1995), with minor modifications (Li et al., 2012). The primers used are listed in Supplemental Table S4.

### Co-immunoprecipitation (CO-IP) assay

For CoIP assays, the vector pMDC83:His-MdFERL6-GFP was used to generate the fusion protein His-MdFERL6-GFP, and the vector pCambia1300:Myc-MdSAMS-YFPn was used to generate the fusion protein Myc-MdSAMS-YFPn. The primers used are listed in Supplemental Table S4. His-MdFERL6-GFP and Myc-MdSAMS-YFPn were co-transformed into apple calli, using co-transformation of His-MdFERL6-GFP and pCambia1300-Myc-YFPn or Myc-MdSAMS-YFPn and pMDC83-GFP as control. The total proteins were extracted 3 days after transformation using extraction buffer (25 mM Tris–HCl, 1 mM EDTA, 10% glycerin, 0.5% Triton X-100, and 1 mM DTT). Protein A+G-Sepharose beads (100 μL, ComWin Biotech) were incubated with 10 μL polyclonal anti-Myc or anti-His antibody (ComWin Biotech) for 2 h at 4°C. An equal amount of anti-Myc antibody-coupled Protein A+G-Sepharose beads was added to total protein samples expressing Myc-MdSAMS-YFPn/His-MdFERL6-GFP and Myc-MdSAMS-YFPn/pMDC83-GFP, and detected with anti-His antibody. An equal amount of anti-His antibody coupled Protein A+G-Sepharose beads was added to total protein samples expressing Myc-MdSAMS-YFPn/His-MdFERL6-GFP and His-MdFERL6-GFP/ pCambia1300-Myc-YFPn and detected with anti-Myc antibody.

### Ethionine sensitivity assay

Apple calli were transfected with *MdFERL6*-OE, *MdFERL6*-AS, and empty vector, and then cultivated on MS medium containing 0, 100, and 200 μM L-ethionine. The growth status of calli was used to reflect the activity of SAM synthase. After a 7-days incubation at 25°C, the weights of the calli were measured and *MdFERL6* and *MdSAMS* expression were determined by qRT-PCR.

### Statistical analysis

Samples were analyzed in triplicate, and the data were noted as the mean + SD. Data were analyzed by Student's *t*-test using SAS software (version 8.1, USA), and the least significant difference at a 0.05 level of probability was used to explore the effect of P input on parameters. *P* < 0.05 was considered as significantly different, *P* < 0.01 was considered as highly significantly different. The statistical significance of differences between samples in **Figure 6C** was tested with ANOVA using the SAS software package 8.02 and the least significant difference (LSD) was used to compare the means.

## Results

### Genome-wide identification of apple *FER*-like genes

**Figure 1:**
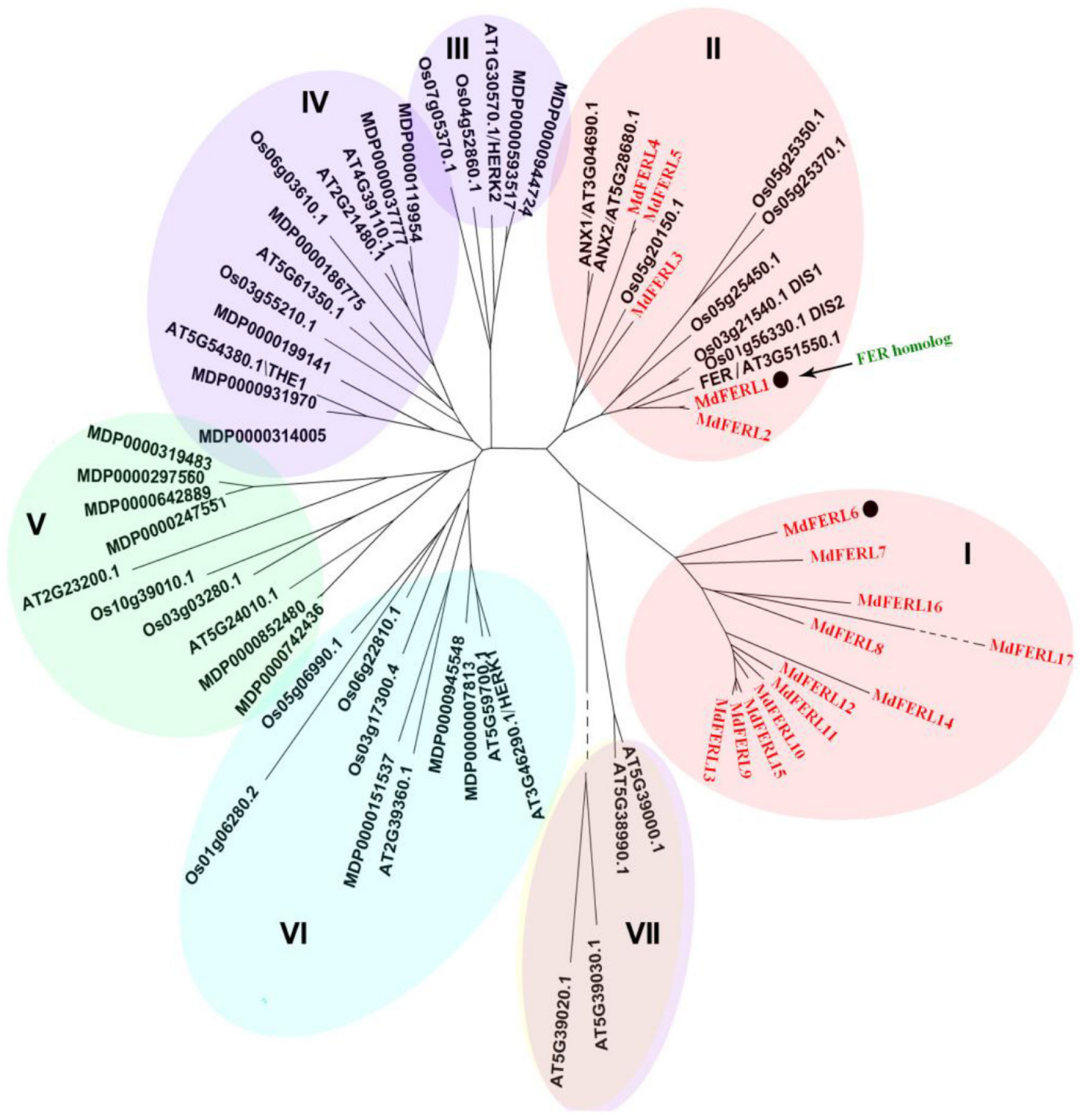
Phylogenetic tree of deduced CrRLK1L amino acid sequences. Phylogenetic analysis of CrRLK1L homologs from various plant species. The phylogenetic tree was constructed using the Neighbor-Joining method in MEGA4.0.2 using 1,000 bootstrap replicates. The numbers at nodes represent bootstrap values. Species name abbreviations are as follows: Md, *Malus* × *domestica*; At, *Arabidopsis thaliana*; Os, *Oryza sativa*.

The FER-like receptor kinases (FERLs) belong to the CrRLK1L family. To identify *FER-like* genes in apple, we first conducted a genome-wide screen for members of the MdCrRLK1L family. The MdCrRLK1L family consists of 34 members, which can be divided into six clades (Figure 1). A comparison of the MdCrRLK1L family with the AtCrRLK1Ls of *Arabidopsis thaliana* and the OsCrRLK1Ls of rice (*Oryza sativa*) indicated that the functionally characterized representative members of AtCrRLK1L, i.e., FERONIA, NAXUR, HERK, and THESEUS, were distributed in different clades, with FER being located in clade II. Interestingly, all members of clade I were apple CrRLK1Ls, suggesting that this group of CrRLK1Ls could be specific to the apple genome. Compared to other genes, the proteins encoded by members of clades I and clade II showed a relatively higher amino acid sequence identity with FER, and hence were designated as FERLs (FERONIA-Like receptor kinase). There were 17 MdFERL members, designated as MdFERL1–17. Clades I and II contained 12 and 5 MdFERLs, respectively. When the MdFERLs were aligned, all members were found to have an extracellular domain containing a malectin domain, which is predicted to bind carbohydrates.

### Expression of *MdFERLs* and ripening-related marker genes during apple fruit development and ripening

**Figure 2:**
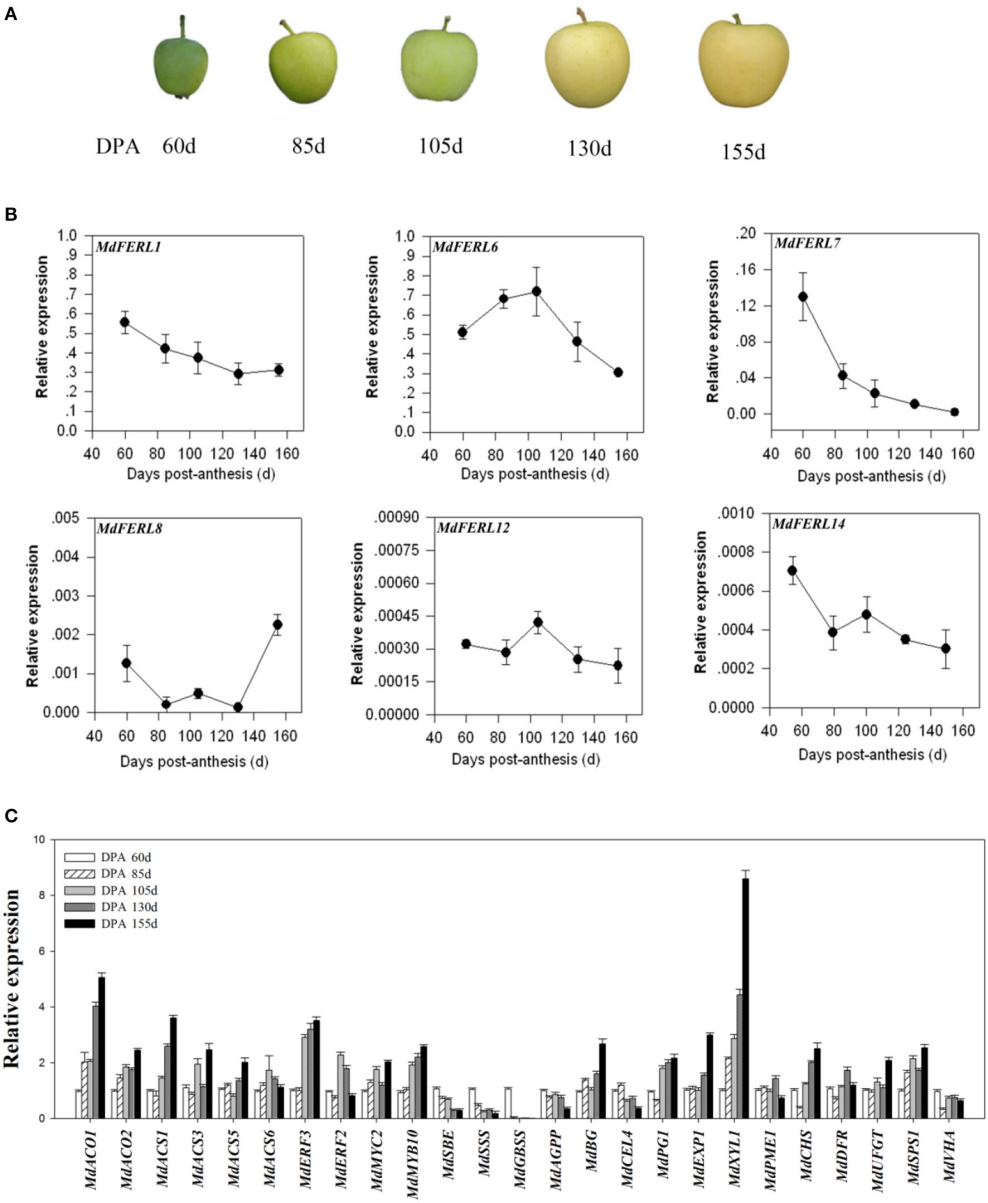
Quantitative reverse transcription (RT-qPCR) analysis of gene expression in apple fruit at different developmental stages. **(A)** Phenotypes of apple fruit at different developmental stages: days post-anthesis (DPA) 60, 85, 105, 130, and 155 days. **(B)** Quantitative reverse transcription (RT-qPCR) analysis of *MdFERL* expression at different developmental stages. **(C)** Quantitative reverse transcription (RT-qPCR) analysis of the expression of ripening-related genes at different developmental stages. *MdACTIN* was used as an internal control. Values are means + SD of three biological replicates.

The expression patterns of *FERL*s during fruit development and ripening are commonly correlated with their corresponding functions. To investigate the potential roles of *MdFERL*s in apple, we first examined their expression patterns in relation to fruit development and ripening. Apple fruit development and ripening occur between anthesis and the time when fruits are ready to be harvested (i.e., during a period of about 160 days) (Figure 2A). A preliminary examination by RT-PCR detected only six *MdFERL* members with a relatively high transcript level in the fruits (Supplemental Figure S1A); therefore, these six genes were further analyzed by RT-qPCR. Two of these genes, *MdFERL1* and *MdFERL6*, were expressed more than 10- to 1,000-fold higher than the others. Strikingly, unlike the expression patterns for the other *MdFERL* members examined, the transcript level of *MdFERL6* was high at the early stages of fruit development, but quickly declined after about 105 DPA (Figure 2B). It has been reported that ethylene production in “golden” apple fruit peaks at around 105 DAF, then remains high during the fruit development and ripening stages (Li et al., 2015). Accordingly, we found that most ethylene biosynthesis and signaling transduction genes, such as *MdACO1, MdACO2, MdACS1, MdACS3, MdERF2*, and *MdERF3*, were significantly upregulated at around 105 DPA and remained high from then onwards (Figure 2C). Comprehensive analysis of the unique expression pattern of *MdFERL6* and of ethylene evolution in apple fruit, suggests that *MdFERL6* regulates ethylene production in apple fruit.

To further characterize the onset of ripening in apple fruit, we evaluated the expression of ripening-related genes such as key transcription factor genes and structural genes involved in pigment accumulation, starch degradation, sugar metabolism, acid metabolism, and fruit softening (Figure 2C). Most ripening-related genes examined, including the softening genes *MdBG, MdEXP1*, and *MdXYL1*, pigment metabolism genes *MdMYB10, MdCHS, MdDFR*, and *MdUFGT*, and the sugar metabolism gene *MdSPS1*, were strongly up-regulated after 105 DPA. Starch biosynthesis genes did not follow this pattern, and were down-regulated at this time point. Collectively, these results suggest that *MdFERL6* is a regulator of ethylene-mediated fruit ripening.

### Expression of *MdFERL*s in response to 1-MCP and ACC treatment

**Figure 3:**
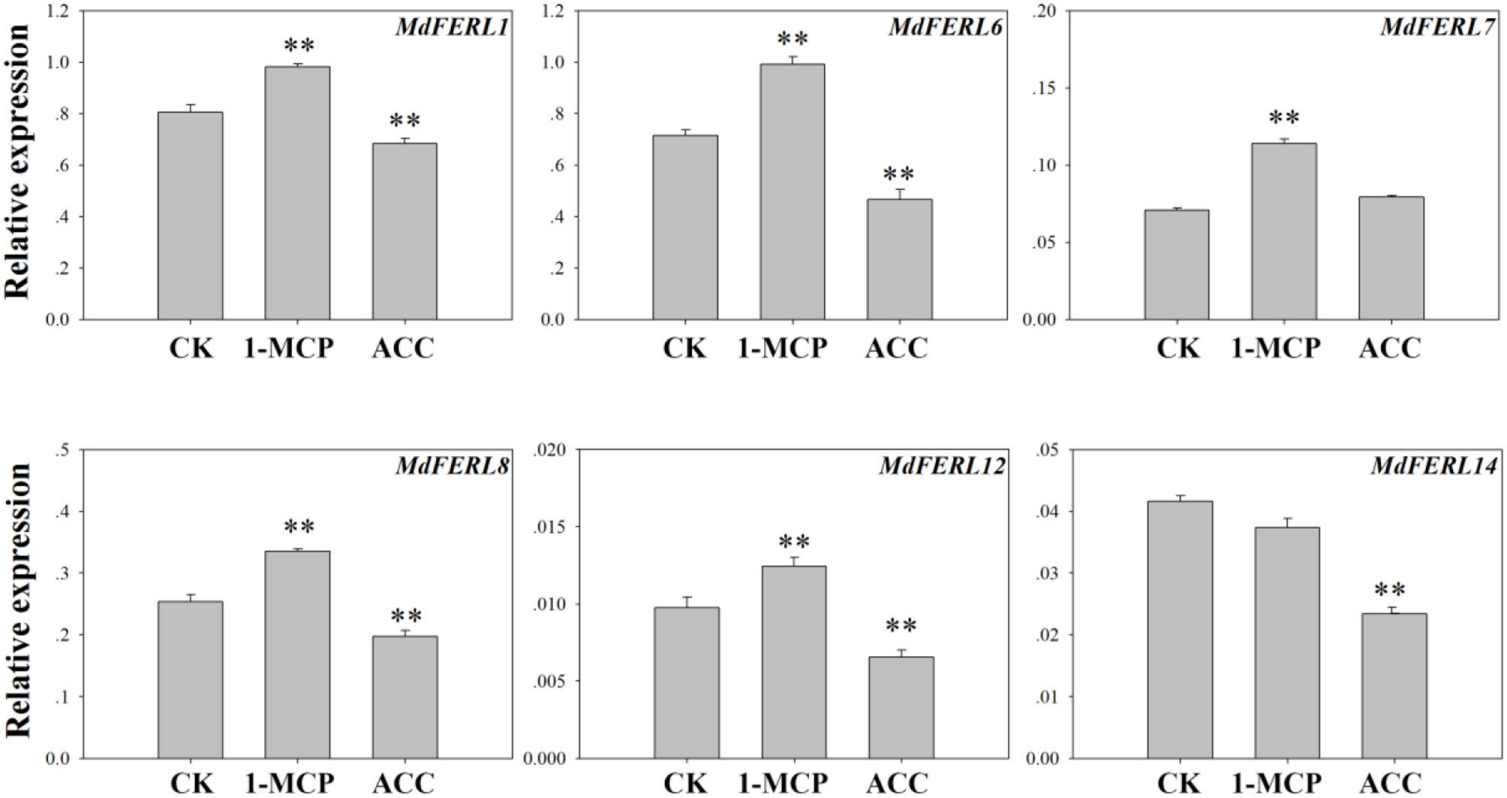
RT-qPCR analysis of *MdFERL* expression in response to 1-MCP and ACC. RT-qPCR was conducted using *MdACTIN* as an internal control. Values are means + SD of three biological replicates. ^**^P < 0.01 (Student's *t*-test), when compared with control values.

To further explore the relationship between FERLs and ethylene in apple fruit, we examined the effects of 1-MCP, an inhibitor of ethylene perception, and ACC (1-aminocyclopropanecarboxylic acid), a precursor of ethylene, on the expression of the six *MdFERL*s evaluated earlier. As shown in Figure 3, *MdFERL1, MdFERL6, MdFERL8*, and *MdFERL12* were upregulated by 1-MCP and suppressed by ACC treatment; however, MdFERL7 could only be induced by 1-MCP and MdFERL14 was only suppressed by ACC (Figure 3).

### Effect of *MdFERL1* and *MdFERL6* manipulation on physiological parameters and molecular events associated with fruit ripening and quality

**Figure 4:**
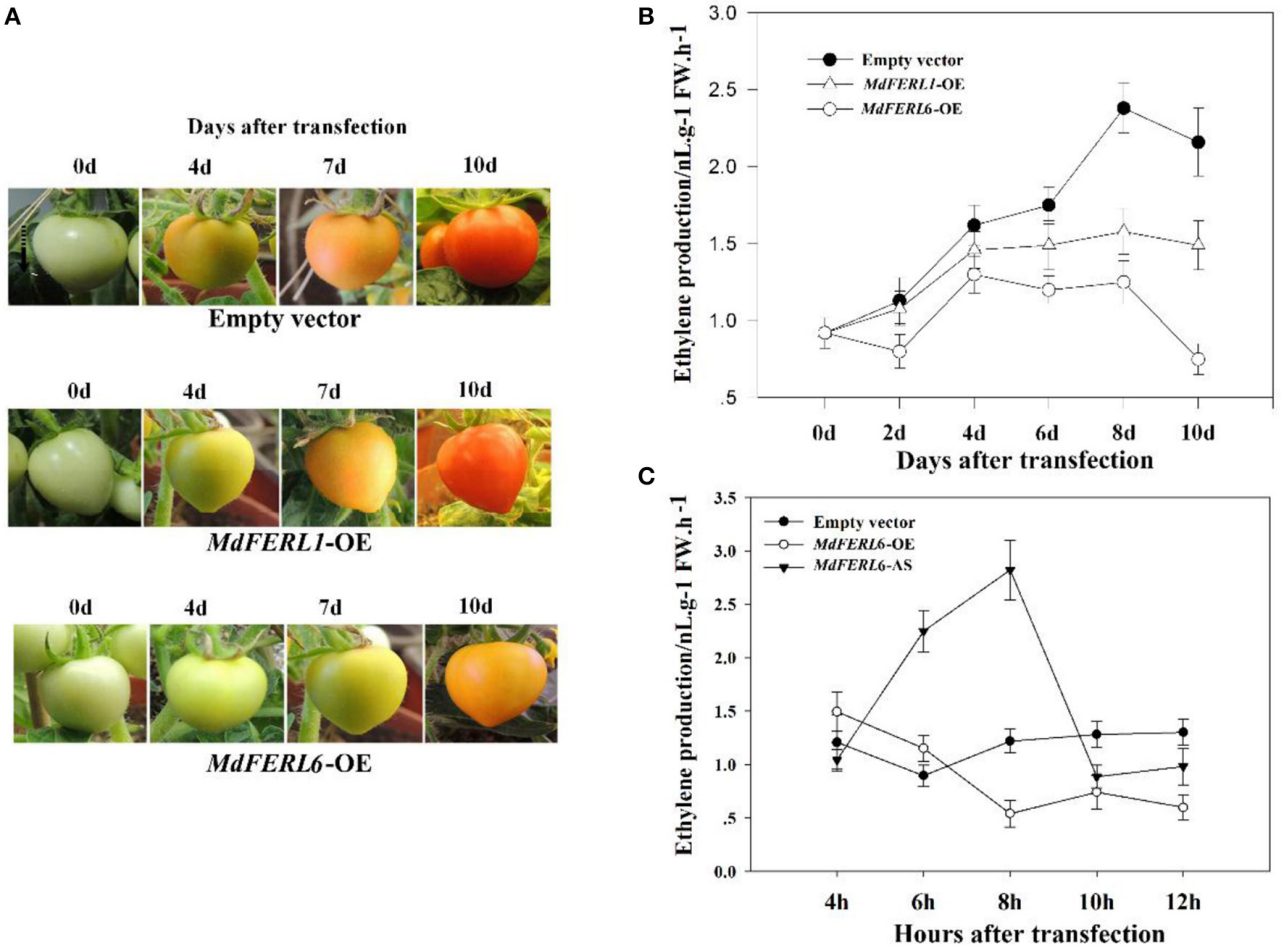
Effect of *MdFERL1* and *MdFERL6* overexpression (OE) and *SlFERL1*-VIGS (virus-induced gene silencing) on transgenic tomato fruit ripening. **(A)** The influence of *MdFERL1*-OE and *MdFERL6*-OE on the time course of tomato fruit development and ripening. **(B)** The effect of *MdFERL1*-OE and *MdFERL6*-OE on tomato fruit ethylene production. **(C)** The effect of *MdFERL6*-OE and *MdFERL6*-AS on apple (*Malus* × *domestica*) callus ethylene production.

As mentioned above, *MdFERL1* and *MdFERL6* have the highest transcript levels in apple fruit among the *FERLs* examined. MdFERL1 has the highest level of amino acid identity with Arabidopsis FER, while MdFERL6 belongs to the group of CrRLK1Ls that appears to be specific to the apple genome. We thus examined the potential roles of *MdFERL1* and *MdFERL6* in fruit ripening and development. Our attempt to manipulate the expression of these genes in a variety of apple species under different conditions failed. However, methods of transgenic manipulation are well established in tomato; therefore, we heterologously expressed *MdFERL1* and *MdFERL6* in tomato fruit. Expressing either *MdFERL1* or *MdFERL6* in tomato fruit delayed ripening in comparison with the control fruit transformed with empty vector (Figure 4A). Since a dramatic increase in ethylene biosynthesis is a critical signal controlling apple and tomato fruit development and ripening, we examined the effects of *MdFERL1* and *MdFERL6* on ethylene production. The heterologous expression of *MdFERL1* or *MdFERL6* both resulted in a significant reduction in ethylene production in comparison with the control (Figure 4B). Notably, the regulatory effect of *MdFERL6* on both fruit ripening (Figure 4A) and ethylene production (Figure 4B) was much stronger than that of *MdFERL1*. These results, in combination with the finding that *MdFERL6* was potentially specific to the apple genome and that the expression pattern of *MdFERL6* was most tightly associated with fruit ripening, prompted us to select this gene for further studies using overexpression and antisense manipulation in apple fruit calli. We found that *MdFERL6* overexpression greatly suppressed ethylene production, and conversely, that antisense expression greatly promoted ethylene production (Figure 4C).

**Figure 5:**
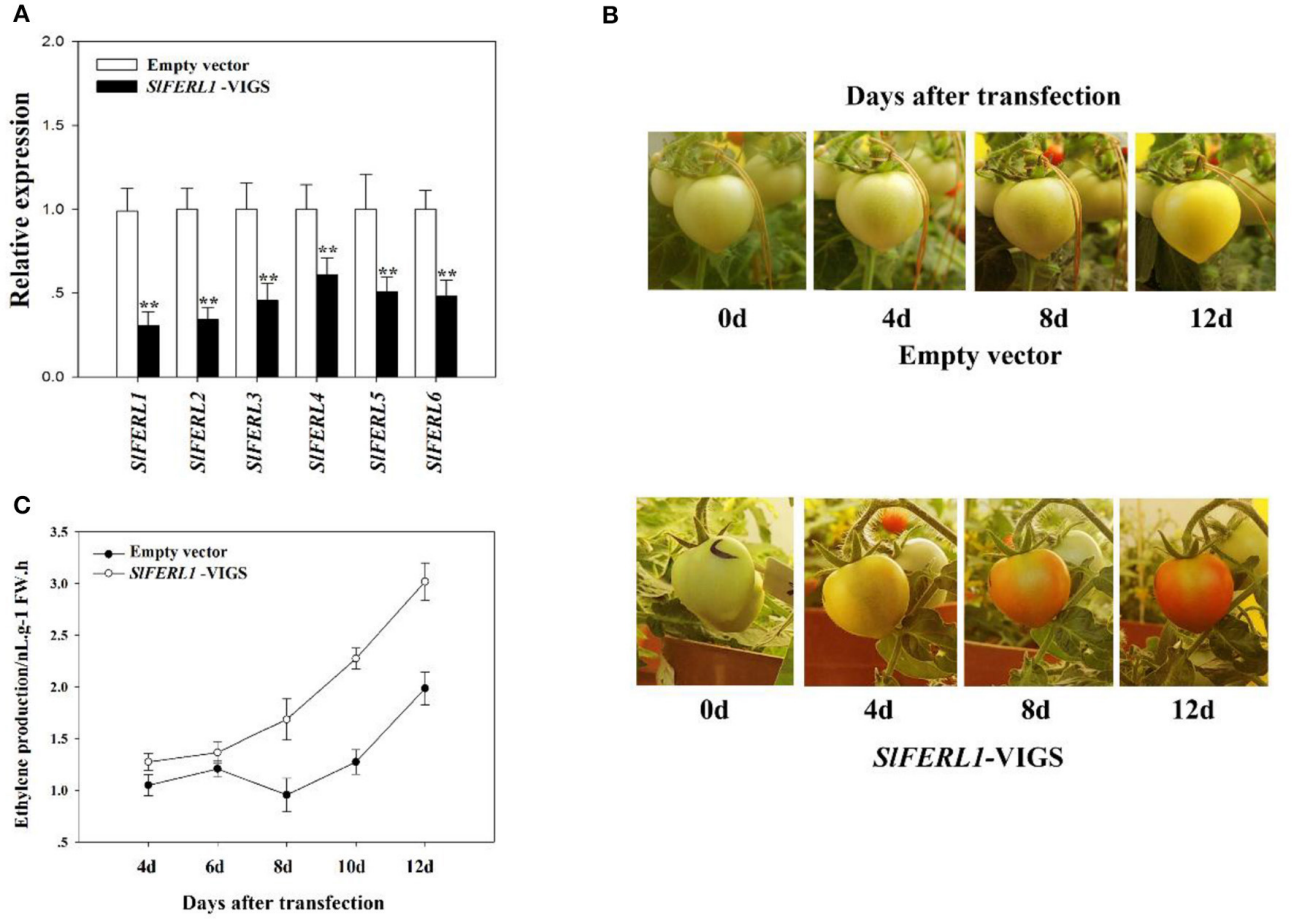
Effect of *SlFERL1*-VIGS (virus-induced gene silencing) on tomato fruit development and ripening. **(A)** The influence of *SlFERL1*-VIGS on the expression of the other *SlFERLs*. RT-qPCR was conducted using *SlACTIN* as an internal control. Values are means + SD of three biological replicates. Double asterisks denote a significant difference at *P* < 0.01 using Student's *t*-test. **(B)** The influence of *SlFERL1*-VIGS on the time course of tomato fruit development and ripening. **(C)** Effect of *SlFERL1*-VIGS on tomato fruit ethylene production.

Since the heterologous expression of *MdFERL1/6* in tomato fruits in combination with the overexpression and antisense expression of *MdFERL6* in apple fruit calli strongly implicated *FERLs* in fruit ripening and ethylene production, we investigated the effect of *FERL* downregulation on fruit ripening. Using VIGS, we investigated the effect of downregulating tomato *FERL* expression on fruit ripening. A search of the tomato genome identified a total of six *FERL*s, designated *SlFERL1–6*, which contained many relatively conserved domains (Supplemental Figure S2). SlFERL1 shared 55.3% amino acid sequence identity with FER, and could be considered a FER homolog; therefore, we selected *SlFERL1* for VIGS manipulation. Unexpectedly, VIGS of *SlFERL1* caused a great decrease in the transcript levels of not only *SlFERL1*, but also of the other five *SlFERL*s (Figure 5A). Furthermore, VIGS of *SlFERL1* greatly promoted fruit ripening (Figure 5B) and ethylene production (Figure 5C).

Fruit ripening involves dramatic changes in a series of physiological parameters, such as pigment content, sugar/acid content, aroma, flavor, and texture. The effect of *FERL* manipulation on fruit ripening described above is reflected by a change in pigment accumulation. To decipher the roles of *FERL*s in fruit development and ripening, we examined their effects on some related physiological parameters. Given that VIGS-mediated silencing of *SlFERL1* downregulated the expression of several other *FERL* members, and importantly, that it affected fruit ripening more strongly than OE, we analyzed various physiological parameters of the VIGS line. We found that *SlFERL1*-VIGS affected several major physiological parameters associated with fruit ripening and quality (Table 1). Strikingly, while *SlFERL1*-VIGS had only a small effect on fructose content, it had a major impact on both sucrose and glucose contents.

**Table 1:** Effects of *SlFERL1*-VIGS (virus-induced gene silencing) on major fruit ripening-related parameters.

**Parameters**	**CK**	**VIGS**	**Notes**
Firmness (kg cm^−2^)	9.067 ± 0.794	7.8 ± 0.825^**^	Cell wall metabolism-related parameter
Flavonoid content (μg g^−1^ FW)	1.782 ± 0.056	1.508 ± 0.079^**^	Polyphenol metabolism-related compounds
Total phenol content (μg g^−1^ FW)	4.37 ± 0.66	3.5 ± 0.31^**^	
Fructose content (mg g^−1^ FW)	15.36 ± 1.613	14.51 ± 1.524	Sugar metabolism-related compounds
Sucrose content (mg g^−1^ FW)	9.925 ± 0.842	7.472 ± 0.694^**^	
Glucose content (mg g^−1^ FW)	33.029 ± 1.964	37.59 ± 2.511^**^	
Total titratable acid content (%)	2.578 ± 0.064	2.786 ± 0.123	Acid metabolism-related parameter
Hydrazinecarboxamide	1.535 ± 0.035	1.665 ± 0.048	Aroma metabolism-related compounds (data are expressed as percentage of the total volatiles)
Acetaldehyde,hydroxy	3.13 ± 0.062	2.3 ± 0.0452	
Silanediol, dimethyl	1.19 ± 0.086	1.855 ± 0.071^**^	
2-Pentenal, (E)	0.175 ± 0.021	0.3 ± 0.056	
2-Pentenal, (E)	1.985 ± 0.079	3.41 ± 0.096^**^	
Oxime-,methoxy-phenyl	0.18 ± 0.056	0.435 ± 0.036^**^	
Heptanal	0.2 ± 0.004	0.11 ± 0.014	
α-Pinene	0.7 ± 0.003	0.53 ± 0.005^**^	
2-Heptenal,(Z)	0.615 ± 0.063	0.575 ± 0.017	
5-Hepten-2-ol, 6-methyl	0.29 ± 0.008	0.275 ± 0.021	
D-Limonene	0.1 ± 0.002	0.87 ± 0.007^**^	
1-Octanol	0.265 ± 0.014	0.36 ± 0.005	
1,6-Octadien-3-ol,3,7-dimethyl	0.13 ± 0.005	0.21 ± 0.009	
Nonanal	0.255 ± 0.002	0.385 ± 0.009	
Methyl Salicylate	1.86 ± 0.042	2.285 ± 0.032^**^	
2-Decenal,(E)	0.11 ± 0.004	0.16 ± 0.007	
2,6-Octadienal,3,7-dimethyl	0.19 ± 0.002	0.15 ± 0.008	
Tetradecane	0.105 ± 0.035	0.13 ± 0.006	
Hexadecane	0.245 ± 0.007	0.275 ± 0.006	
Phenol,2,4-bis(1,1-dimethylethyl)	0.008 ± 0.001	0.1 ± 0.003^**^	
Dodecanoic acid, ethyl ester	0.07 ± 0.002	0.23 ± 0.006^**^	

*The SlFERL1-VIGS construct was transfected into tomato at 18 days post-anthesis. At 12 days after transfection, fruits were detached and analyzed. Values are means ± SD of two samples (each sample includes five fruits). Asterisks denote significant differences compared with the control sample (i.e., VIGS-C) at P < 0.01, using Student's t-test. C, control; FW, fresh weight*.

**Figure 6:**
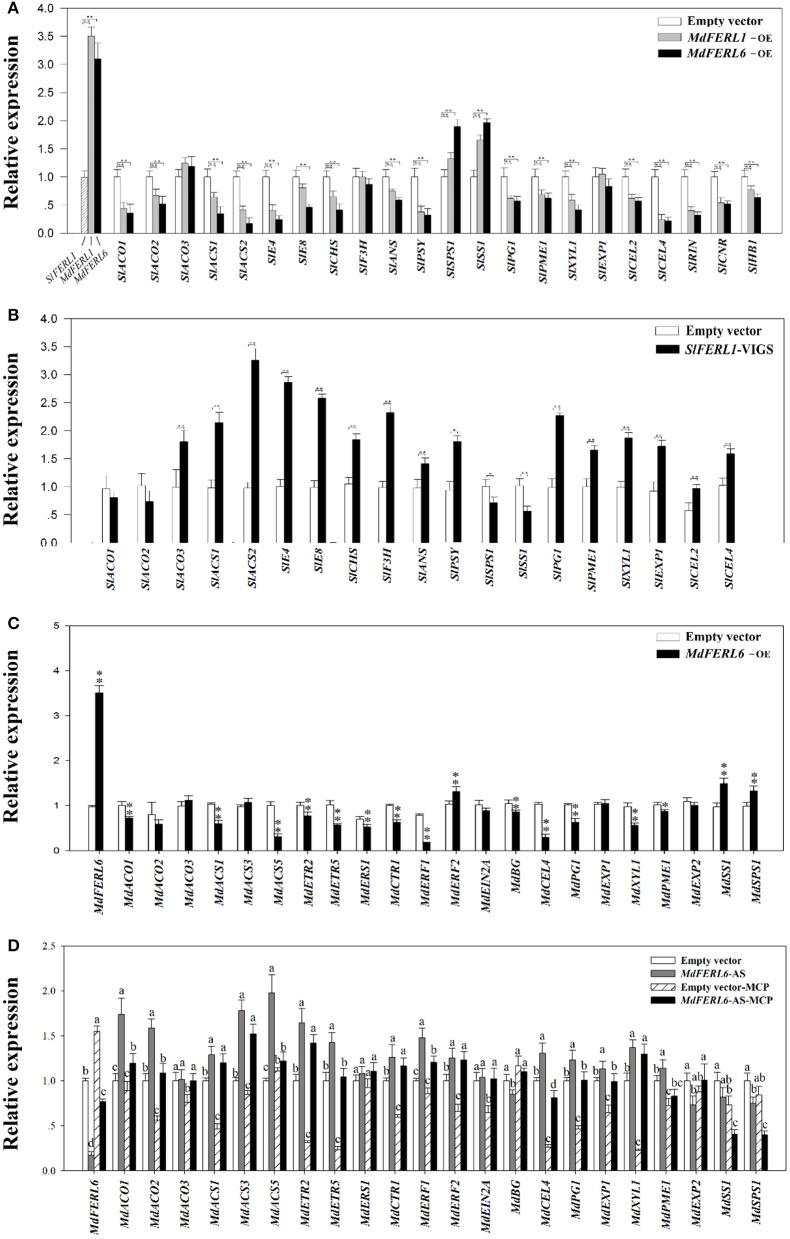
Effect of *MdFERL1, SlFERL1*, and *MdFERL6* on the expression of ripening-related genes. **(A)** Effect of *MdFERL1* and *MdFERL6* on the expression of tomato ripening-related genes. The *MdFERL1*-OE (overexpression) and *MdFERL6*-OE constructs were injected into tomato fruits at 18 DPA and gene expression was analyzed 10 days after transfection. Control samples were transfected with the empty vector (pCambia1304). RT-qPCR was conducted using *SlACTIN* as an internal control. Values are means + SD of three biological replicates. Double asterisks denote a significant difference at ^**^P < 0.01 and ^*^P < 0.05 using Student's *t*-test. **(B)** Effect of *SlFERL1*-VIGS (virus-induced gene silencing) on the expression of tomato ripening-related genes. The VIGS constructs were injected into fruits at 18 DPA, and gene expression was examined 12 days after transfection, using RT-qPCR. *SlACTIN* was used as the internal control. Values are means + SD of three biological replicates. Double asterisks denote a significant difference at ^**^P < 0.01 and ^*^P < 0.05 using Student's *t*-test. **(C)** Effect of *MdFERL6*-OE on the expression of ripening-related genes in apple calli. RT-qPCR was conducted using *MdACTIN* as an internal control. Values are means + SD of three biological replicates; different lowercase letters represent significant differences based on ANOVA (*P* < 0.05). **(D)** Effect of *MdFERL6*-AS and *MdFERL6*-AS combined with 1-MCP on the expression of ripening-related genes in apple calli. RT-qPCR was conducted using *MdACTIN* as an internal control. Values are means + SD of three biological replicates; different lowercase letters represent significant differences based on ANOVA (*P* < 0.05).

To further probe the mechanisms by which *MdFERL*s influence fruit development and ripening, we examined the altered expression of important fruit development genes following the manipulation of the *MdFERL*s. Given that ethylene production was greatly affected by the changes in the expression of the *MdFERL*s, we focused on genes associated with ethylene biosynthesis and signaling responses, such as *ACO, ACS, ERF, E4*, and *E8*. We also examined the expression of several marker genes of fruit ripening and quality, such as *CHS, F3H, ANS*, and *PSY*, which function in pigment metabolism, *SS* and *SPS*, which are involved in fruit sugar metabolism (fruit quality), *PG, PME, XYL*, and *EXPs*, which function in cell wall metabolism (fruit texture), and *RIN, CNR*, and *HB1*, which encode ripening-related transcription factors in tomato. Heterologous expression of *MdFERL1* and *MdFERL6* in tomato fruits significantly suppressed the expression of most of the genes examined, except for *SPS1* and *SS*, which were up-regulated rather than suppressed, and *EXP1*, which was not altered (Figure 6A). In accordance with these observations, VIGS of *SlFERL1* resulted in an increase in the transcript levels of most of the genes examined, particularly *ACS, E4*, and *E8*, but the expression of *SS* and *SPS1* decreased, while no change was observed for *ACO1* or *ACO2* (Figure 6B). To further reveal how *MdFERL6* regulates fruit ripening and to identify its precise role in ethylene-mediated fruit ripening, we examined the expression of various genes involved in the regulation of ethylene biosynthesis, ethylene signaling transduction, and fruit ripening in apple fruit calli in which the levels of *MdFERL6* had been manipulated. Overexpression of *MdFERL6* caused a dramatic decrease in the expression of the ethylene biosynthesis genes *MdACO1, MdACS1, MdACS5;* ethylene signal transduction genes *MdCTR1, MdETR2*, and *MdETR5;* and fruit ripening-related genes *MdCEL4, MdPG1*, and *MdXYL1* (Figure 6C). By contrast, antisense expression of *MdFERL6* caused a great increase in the expression of these genes (Figure 6D). Furthermore, the expression of most of the analyzed genes was suppressed by 1-MCP treatment. Interestingly, *MdACO2, MdACS5, MdETR5, MdERF1, MdCEL4, MdPG1, MdEXP1*, and *MdPME1* expression was less sensitive to 1-MCP treatment in apple calli harboring the *MdFERL6* antisense construct (*MdFERL6*-AS) than in non-transgenic control calli treated with 1-MCP, while the expression of *MdSS1* and *MdSPS1* was more sensitive to 1-MCP treatment, suggesting that all of these genes are regulated by ethylene through MdFERL6-mediated pathways. However, compared to non-treated *MdFERL6*-AS calli, the expression of *MdACS1, MdACS3, MdETR2, MdCTR1, MdERF2, MdXYL1*, and *MdEXP1* was not significantly altered by 1-MCP, suggesting that MdFERL6 also regulates fruit ripening via an ethylene-independent mechanism or that MdFERL6 functions downstream of the examined signaling proteins (e.g., MdCTR1 and MdERF2; Figure 6D). The intricate roles of MdFERL6 in the regulation of fruit ripening and ethylene signal transduction merit further investigation.

### MdFERl1 and MdFERL6 physically interact with MdSAMS

**Figure 7:**
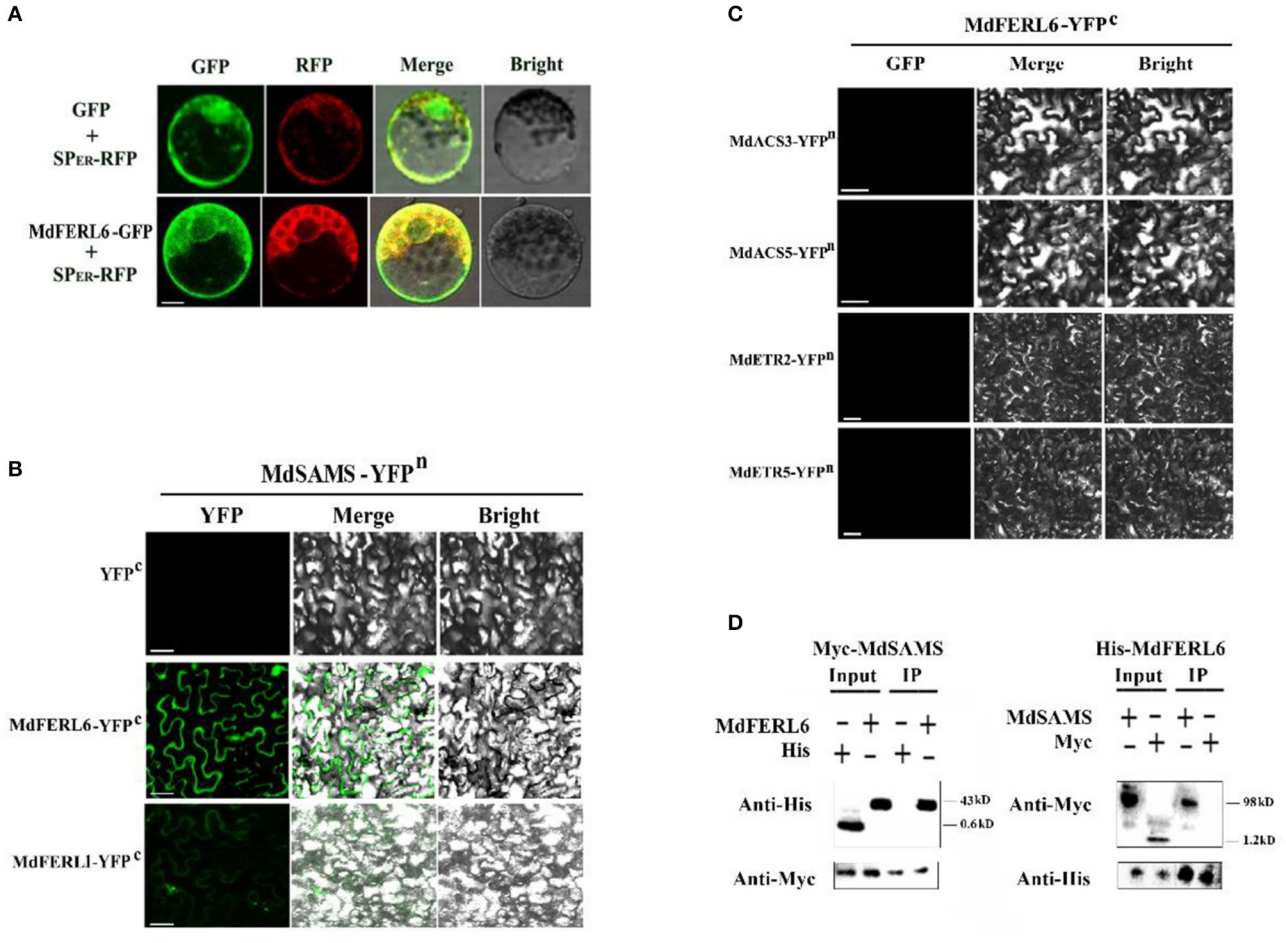
Subcellular localization of MdFERL6 and its physical interaction with proteins involved in ethylene production and signal transduction. **(A)** Subcellular localization of MdFERL6. pMDC83-*MdFERL6* was transformed into maize (*Zea mays*) protoplasts, and fluorescence was observed by confocal microscopy. Bars = 10 μm. **(B)** Bimolecular fluorescence complementation (BiFC) analysis of the physical interaction between MdFERL1 or MdFERL6 and MdSAMS, fused with the C-, C-, and N-terminus, respectively, of yellow fluorescent protein (YFP; designated as YFPc or YFPn, respectively). Different combinations of the fused constructs were co-transformed into tobacco (*Nicotiana tabacum*) cells, and visualized using confocal microscopy. YFP and bright-field were excited at 488 nm and 543 nm, respectively. Bars = 20 μm. **(C)** BiFC analysis of the physical interaction between MdFERL6 and MdACS3, MdACS5, MdETR2, and MdETR5. MdFERL6 was fused to the C-terminus of YFP, while the other proteins were fused to the N-terminus of YFP. **(D)** Co-immunoprecipitation (Co-IP) assay of the interaction between MdFERL6 and MdSAMS in apple calli. MdFERL6 was fused with GFP/His using the pMDC83 vector, and MdSAMS was fused with Myc using the pCambia1300 vector. (The molecular weight of Myc-MdSAMS is 43 kD, Myc is 1.2 kD, His-MdFERL6 is 98 kD, and 6 × His is 0.6 kD).

Given that MdFERL1 and MdFERL6 were shown to regulate ethylene production as well as ethylene responses, we further tested the possibility that these enzymes physically interact with key enzymes in the ethylene biosynthesis pathway and the signaling proteins involved in the ethylene response. The ethylene receptor ETRs are central proteins in ethylene signal transduction, and are localized to the endoplasmic reticulum. Interestingly, in addition to its localization to the membrane, MdFERL6 was also found to localize to the ER (Figure 7A). We thus tested the possibility that MdFERL6 interacts with ethylene receptors, using a BiFC assay. We also tested whether three well-characterized key enzymes in the ethylene biosynthesis pathway, SAMS, ACS, and ACO, could interact with MdFERL1 and MdFERL6. While fluorescence was not observed in the control (combination of empty vectors) or in the combinations of MdFERL6 and MdACS and of MdFERL6 and MdACO, strong fluorescence appeared when MdSAMS-YFP^n^ was combined with MdFERL1 and MdFERL6 (Figures 7B,C). Furthermore, MdFERL6 did not appear to interact with the ethylene receptor MdETRs. The interaction between MdFERL1/6and SAMS further indicate that MdFERL1 and MdFERL6 modulate ethylene production during fruit development and ripening. To test whether MdFERL6 could interact with MdSAMS in apple calli, we conducted a co-immunoprecipitation (Co-IP) assay. As shown in Figure 7D, the Myc-MdSAMS protein complex (43 kD) was co-immunoprecipitated by His-MdFERL6 with anti-His antibody, and the His-MdFERL6-GFP complex (98 kD) was co-immunoprecipitated by Myc-MdSAMS with anti-Myc antibody. These results suggest that MdFERL1 and MdFERL6 may interact *in vivo*.

### Ethionine activity assay in *MdFERL6*-OE and *MdFERL6*-AS apple calli

**Figure 8:**
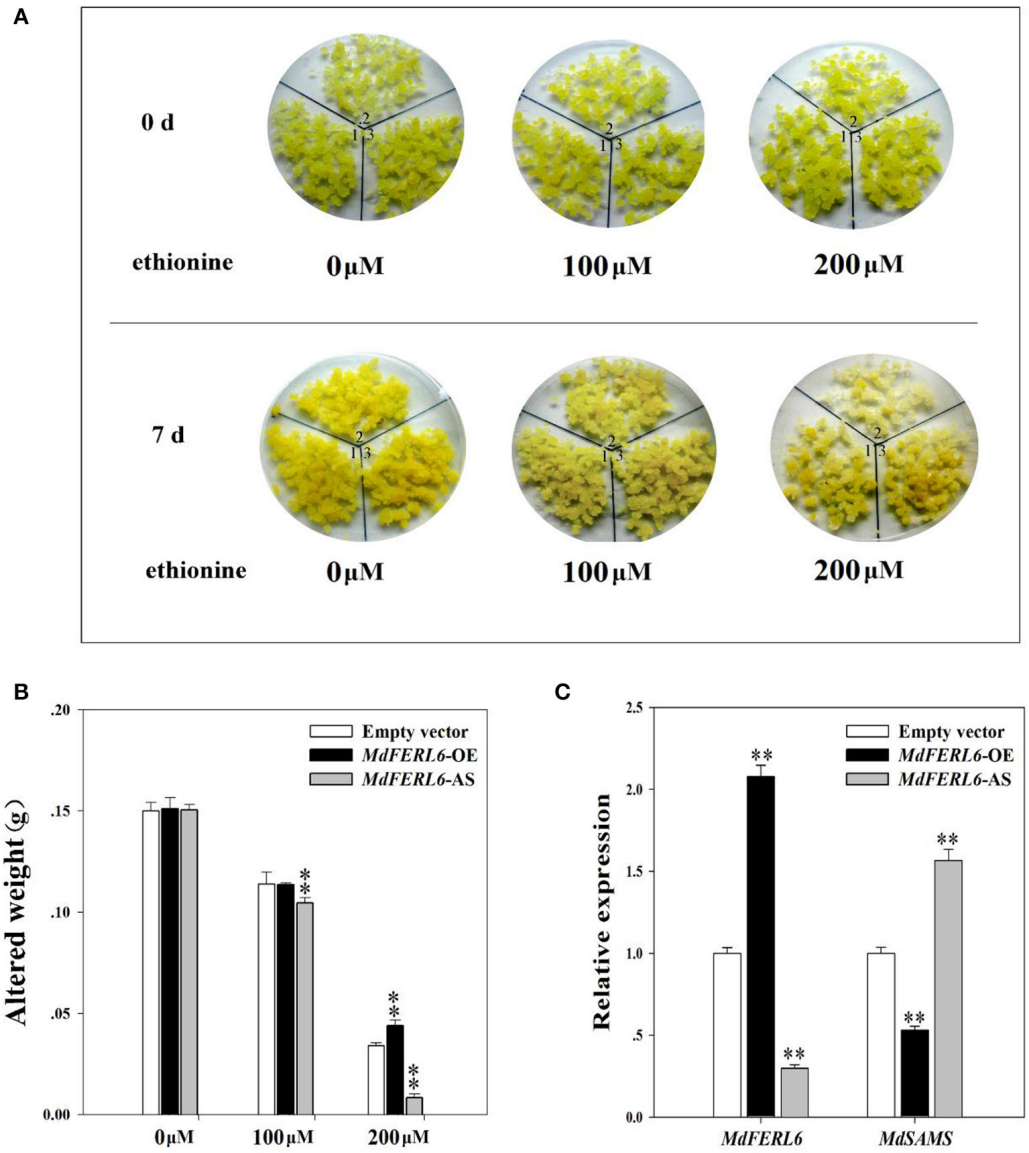
Ethionine activity assay. **(A)** Ethionine altered the growth status of apple calli. Apple calli transfected with empty vector (1); *MdFERL6*-AS (2); and *MdFERL6*-OE (3). **(B)** Measurement of calli weight. Transfected apple calli were weighed before treatment (0 day, and were then cultivated with ethionine for 7 days. The increase in weight between 0 and 7 days is indicated on the y-axis. Values are means + SD of three biological replicates. Single asterisks denote a significant difference at *P* < 0.05 using Student's *t*-test; double asterisks denote a significant difference at *P* < 0.01 using Student's *t*-test. **(C)** Gene expression of *MdFERL6* and *MdSAMS* were detected in apple calli cultivated in 200 μM ethionine for 7 days. Values are means + SD of three biological replicates. Single asterisks denote a significant difference at *P* < 0.05 using Student's *t*-test; double asterisks denote a significant difference at *P* < 0.01 using Student's *t*-test.

Having demonstrated that MdFER6 physically interacts with MdSAMS in apple, we further examined the roles of the MdFERL-SAMS protein complex in ethylene production by performing an ethionine activity assay. Ethionine is a toxic analog of methionine (Met). When cultivated with ethionine, plants with higher SAMS activity absorb more ethionine, resulting in toxic symptoms. Thus, plant growth status can be used as an indicator of SAMS activity (Mao et al., 2015). As shown in Figure 8, when cultivated in the presence of ethionine, the growth status of MdFERL6-OE and MdFERL6-AS transgenic calli was altered. *MdFERL6*-AS transgenic calli were more sensitive than control calli to ethionine treatment. After 7 days of cultivation, *MdFERL6*-AS transgenic calli had become pale and gained less weight than control calli, suggesting that SMAS has higher activity in *MdFERL6*-AS transgenic calli than in the control. Furthermore, after 7 days of cultivation, *MdFERL6*-OE transgenic calli had become dark yellow and, for the 200 μM treatment, had gained more weight than the control, indicating that overexpression of *MdFERL6* suppresses SAMS activity (Figures 8A,B). Furthermore, the expression of *MdSAMS* was increased in *MdFERL6*-AS and repressed in *MdFERL6*-OE, suggesting that MdFERL6 affects SAM synthesis through both activity level and transcription level (Figure 8C).

## Discussion

The first receptor-like protein kinase with a carbohydrate-binding domain, malectin, was identified from *Catharanthus roseus*, and thus named CrRLK1 (Schulze-Muth et al., 1996). Since then, CrRLK1-like receptor kinases (CrRLK1Ls) have increasingly attracted the interest of plant biologists. The genome size of rice is nearly four times that of *Arabidopsis thaliana* (Hong et al., 1997; Arabidopsis Genome Initiative, 2000), yet the number of CrRLK1Ls in rice (20 genes) is only slightly larger than that in Arabidopsis (17 genes; Kessler et al., 2010; Haruta et al., 2014). By contrast, the genome size of apple is only slightly larger than that of rice (Hong et al., 1997; Velasco et al., 2010), yet in the present study, we found that the CrRLK1L family in apple contains 34 members, and is therefore much larger than its counterpart in Arabidopsis and rice. In the context of plant morphology and anatomy, one of the major differences between apple and Arabidopsis or rice is the production of fleshy fruit, and accordingly, we were interested in determining whether the large difference in CrRLK1L family size between these species is correlated with fruit development. Our phylogenetic tree of CrRLK1L members from three species contained seven clades, five of which contained members from all three species. Clade I was not only specific to apple, but also contained more MdRLKs (12 members) than the other clades, while clade II contained the AtFER homolog, MdFERL1. We thus focused on MdFERLs in clades I and II in the present study.

Apple is one of the most popular and economically important fruit crops worldwide, and knowledge of the molecular mechanisms underlying apple fruit development and ripening could have beneficial applications in the apple industry. Difficulties in apple transgenesis, such as the long time required to obtain transgenic fruit and the lack of an efficient transformation system, have meant that molecular studies of apple fruit development and ripening have been limited, with most studies being performed in a tomato model. Despite extensive efforts, we failed to develop a transient transgenic system that could be successfully used for gene manipulation in apple fruits. In the present study, we examined apple fruit development and ripening using three approaches: (1) the heterologous expression of *MdFERL1* and *MdFERL6* in tomato fruit, (2) VIGS-mediated manipulation of *SlFERL1* in tomato fruit, and (3) overexpression and antisense transformation of the apple genes in apple fruit calli. Heterologous expression of *MdFERL1* and *MdFERL6* in tomato fruits delayed fruit ripening, while downregulation of *SlFERL1* expression dramatically promoted tomato fruit ripening. The two systems corroborate each other, collectively demonstrating the pivotal roles of *MdFERL1* and *MdFERL6* in fruit development and ripening. Strikingly, when compared with the heterologous expression experiment, the effect of *SlFERL1* VIGS on fruit ripening was much stronger. To elucidate this response, we examined the effect of *SlFERL1* VIGS on the transcript levels of the other *SlFERLs* we identified in the tomato genome, and found it to cause a significant decrease in the expression of all five related genes. This implies that multiple *SlFERL* genes play important roles in fruit development and ripening. In this study, we identified 17 *MdFERLs* in the apple genome, and demonstrated that two of these members, *MdFERL1* and *MdFERL6*, regulate fruit development and ripening. To fully understand the roles of *MdFERL*s in these processes, the contribution of the remaining *MdFERL*s should be examined.

Since its identification as an essential regulator of female fertility (Huck et al., 2003; Rotman et al., 2003), FER has been found to control a series of different developmental and biological processes. Besides its critical roles in fertilization (Escobar-Restrepo et al., 2007; Duan et al., 2014; Ngo et al., 2014; Liu et al., 2016), it has also been implicated in the regulation of cell elongation (Guo et al., 2009), root hair growth (Duan et al., 2010), seed size (Yu et al., 2014), plant defense (Keinath et al., 2010; Kessler et al., 2010), and mechanosensing and calcium signaling (Shih et al., 2014). In the context of metabolism, FER was found to be a multifunctional regulator of starch accumulation (Yang et al., 2015) and sugar metabolism (Yeats et al., 2016; Pu et al., 2017). Recently, FER has been implicated in the cross-talk signaling of various phytohormones, including in ABA (Yu et al., 2012; Chen et al., 2016), auxin (Duan et al., 2010), BR, and ethylene (Guo et al., 2009; Deslauriers and Larsen, 2010) signaling. FER may also interact with SAM/SAMS, thereby suppressing ethylene production in Arabidopsis (Mao et al., 2015). We found that MdFERL6 could physically interact with MdSAMS, leading to changes in *MdSAMS* expression and MdSAMS activity in apple (Figures 7, 8). Phosphorylation site analysis identified five sites in MdSAMS, 134T, 153T, 250T, 271S, and 284Y, that can be potentially be phosphorylated by kinases (http://kinasephos.mbc.nctu.edu.tw/index.php). However, the mechanism by which MdFERL6 and MdSAMS interact, including the involvement of phosphorylation, remains to be elucidated. A recent study showed that a FERONIA-like kinase (MDP0000493959), which was identified as MdFERL12 in our study, was transcriptionally downregulated by ethylene during postharvest ripening and senescence of apple fruit (Zermiani et al., 2015). We found that *MdFERL12* was expressed at lower levels than *MdFERL1* and *MdFERL6* in apple fruit (Figure 3, Supplemental Figure S1B); however, *MdFERL12* could be induced by 1-MCP and other ripening-related signals such as mannitol, PEG, and high temperature (Supplemental Figure S3), suggesting that FERLs have diverse functions in regulating ethylene production and signal transduction, and that their mechanisms need to be further revealed.

Although FER has been extensively studied as described above, a potential role of FERLs in fruit development has not been reported. The present study demonstrates that FERLs are critical regulators of fruit development and ripening, further indicating the importance of FERLs as universal regulators of plant growth and development. In contrast to Arabidopsis, which has just one FER gene, the apple genome contains 14 FERLs. If each individual MdFERL member controls multiple developmental or biological processes, the mechanisms by which these proteins collectively or synergistically regulate apple plant growth and development may be of great significance, and should be thoroughly investigated.

Ethylene has long been known to be the critical signal controlling climacteric fruit ripening (Biale, 1964; Burg and Burg, 1965; Alexander and Grierson, 2002; Bram et al., 2015). One of the important features of ethylene signaling is its large increase in production during fruit ripening; therefore, any cellular internal factor controlling ethylene production should be investigated as a critical regulator of climacteric fruit development and ripening. It has been well established that there are three key enzymes in the ethylene biosynthesis pathway; SAM synthase, ACC Synthase (ACS), and ACC oxidase (ACO). SAM synthase catalyzes the first step in the ethylene biosynthesis pathway, namely the formation of S-adenosylmethionine from methionine and ATP. ACS catalyzes the formation of ACC from S-adenosylmethionine. In the present study, we revealed that both MdFERL1 and MdFERL6 physically interact with SAMS, but not with ACS or ACO. The present study suggests that multiple FER-like protein kinases physically interact with SAMS; such a variety of interactions is not possible in Arabidopsis, as it contains just one member of this family. The finding that different MdFERLs jointly regulate the same biological process reflects the significance of the existence of so many FERLs in the apple genome. As ACC is the immediate precursor of ethylene, the rate-limiting step of ethylene biosynthesis is, under most conditions, considered to be the conversion of S-adenosylmethionine to ACC by ACS (Wang et al., 2002). Thus, the regulation of ethylene synthesis via SAMS rather than ACS or ACO suggests that other rate-limiting steps exist in this pathway. In a previous study, we found that the amount of ABA that accumulated under drought stress was controlled by enzymes that function in the early stages of ABA biosynthesis, rather than by 9-cis-epoxycarotenoid dioxygenase (NCED), an enzyme that catalyzes the production of xanthin, the immediate precursor of ABA (Ren et al., 2007). Once ABA starts to accumulate, its immediate precursors are rapidly exhausted, to the extent that the early stages in the pathway become the rate-limiting steps that determine how much ABA accumulates, rather than the NCED-catalyzed step. A similar mechanism operates in ethylene production during climacteric fruit ripening; the regulation of ethylene production by FER-like protein kinases occurs at an early stage of ethylene biosynthesis (at the step catalyzed by SAMS), rather than at the downstream enzymes ACS and ACO.

In climacteric fruits, ethylene production occurs via two systems, the origins of which are key to our understanding of the mechanisms regulating fruit development and ripening. The first system is basal low rate of ethylene production, which occurs until fruit ripening commences, while the second system generates the large increase in ethylene production during fruit ripening (McMurchie et al., 1972; Hoffman and Yang, 1980; Brady, 1987). Given that both MdFERL1 and MdFERL6 suppress ethylene production, it is reasonable to propose that MdFERL1 and MdFERL6 are implicated in the first system, acting to inhibit ethylene biosynthesis before fruit ripening occurs. Nevertheless, the transcript levels of both *MdFERL1* and *MdFERL6* declined as apple fruit growth and ripening progressed, suggesting that MdFERL1 and MdFERL6 are likely associated with the regulation of the ethylene production spike during ripening. Notably, in contrast to the continual decline in *MdFERL1* expression throughout the process of fruit set to ripening, the transcript level of *MdFERL6* remained high until about 100 DPA, after which it decreased dramatically. Compared with *MdFERL1, MdFERL6* had a stronger effect on fruit ripening in transgenic tomatoes, suggesting that it is the decline of the *MdFERL* transcripts at the later development stages that is most important for the regulation of fruit ripening. On the other hand, the different patterns of *MdFERL1* and *MdFERL6* expression implies that ethylene production is tightly modulated throughout the process, from fruit set to ripening; individual *MdFERL*s may play different roles in the two phases of ethylene production, and they collectively or synergistically determine the spatio-temporal changes in ethylene content.

In summary, we found that FERLs in apple can be categorized into two groups; some share a relatively high level of amino acid sequence identity with the FERLs in both Arabidopsis and rice, while others are less similar and appear to be specific to the apple genome. MdFERL1 and MdFERL6 belong to the first and second of these groups, respectively, and were the most highly expressed of all the *MdFERL*s in developing fruit. The following four findings support the notion that *MdFERL1* and *MdFERL6* regulate fruit ripening: (1) heterologous expression of *MdFERL1* and *MdFERL6* delayed fruit ripening and suppressed ethylene production in tomato; (2) overexpression and antisense silencing of *MdFERL6* suppressed and promoted ethylene production, respectively; (3) VIGS of*SlFERL1* promoted fruit ripening and ethylene production; and (4) MdFERL1 and MdFERL6 were capable of physically interacting with the key ethylene biosynthesis enzyme, SAMS. As MdFERL6 and MdFERL1 suppress ethylene production during fruit development and ripening, their high transcription levels in the early stages of fruit growth followed by their dramatic decreases (especially for *MdFERL6*) during fruit ripening imply that they contribute to the regulation of ethylene production throughout development; i.e., they maintain ethylene at relatively low levels during the early developmental stages, and boost ethylene levels when fruit ripening commences. MdFERL1 and MdFERL6 therefore function as negative regulators of climacteric fruit ripening. This work has, for the first time, demonstrated that FER-like protein kinases are implicated in fleshy fruit development and ripening, providing new insights into the molecular basis of these processes. Nevertheless, this is a preliminary study; more research is required to elucidate the molecular recognition and signaling cascades of MdFERLs during fruit development and ripening.

## Author contributions

MJ and PD, performed most of the experiments; ND, QZ, and SX, contributed to some of the experiments; LW, YZ, WM, and JL, provided technical assistance; BL, designed the experiments and contributed to the date analysis; WJ, conceived the project, supervised the experiments, and complemented the writing.

### Conflict of interest statement

The authors declare that the research was conducted in the absence of any commercial or financial relationships that could be construed as a potential conflict of interest.
