# Supplementary material for: Two FERONIA-Like Receptor Kinases Regulate Apple Fruit Ripening by Modulating Ethylene Production
Source: Front Plant Sci. 2017 Aug 10;8:1406. doi: 10.3389/fpls.2017.01406 (PMC5554343; doi:10.3389/fpls.2017.01406)
Supplement: Supplementary file 1 [file DataSheet1.docx]

**Supplementary Table S1**: Primers used to amplify *MdFERL1*, *MdFERL6,* and *SlFERL1* coding sequences.

| **Gene** | **Genbank accession number** | **Forward primer (5’-3’)** | **Reverse primer (5’-3’)** |
| --- | --- | --- | --- |
| *MdFERL1* | KY435591 | GGTACCATGATTATGAACACTGCTGGGTTTAT | GAATTCACGTCCTTTAGGGTTCATGATTTGTG |
| *MdFERL6* | KY435598 | GGTACC ATGAATCCCAATCTTGCTCATC | GAATTC TCACCTTCCATTGGGATCG |
| *SlFERL1* | KY435602 | CTCGAGGCTTCTTGGGGTGGGTGGTTTTG | CCCGGGGTTCAGAGCTGGCCGAGCACAC |

**Supplementary Table S2**: Reverse transcription polymerase chain reaction (RT-qPCR) primers used to detect *MdFERLs* expression.

| **Gene** | **Genbank accession number** | **Forward primer (5’-3’)** | **Reverse primer (5’-3’)** |
| --- | --- | --- | --- |
| *MdFERL1* | KY435591 | GCGACGACTCTGACGGATTG | TGTGAGAACACGGCACTTGG |
| *MdFERL6* | KY435598 | GTAGGCAGCATAAACCCG | CATTCCAGTGTCCCGATT |
| *MdFERL7* | KY435592 | CAACTCCGCCACCGTCAT | AACCCGAAATCCGAAACC |
| *MdFERL8* | KY435597 | TCTACATAGCCAATCA | CCAAGGACCTACTAAC |
| *MdFERL12* | KY435594 | GGACTCGCCGCCAATCTA | CAATATCTCCATCCCAAT |
| *MdFERL14* | KY435596 | CTCTTCTTTACCGTCCGATTTGT | TAAGTTGTGCGAGCAGTTTGATT |

**Supplementary Table S3：**RT-qPCR primers used to detect the expression of ripening-related genes.

| **Gene** | **Full Name** | **Forward primer (5’-3’)** | **Reverse primer (5’-3’)** | |
| --- | --- | --- | --- | --- |
| *SlACO1* | [1-Aminocyclopropane-1-carboxylic Acid Oxidase](http://fjour.blyun.com/views/specific/3004/FJourDetail.jsp?dxNumber=165198978170&d=51DC9D17B8D210C15454F6F09E223FBC&s=ACO1)1 | ACAAACAGACGGGACACGAA | | CTCTTTGGCTTGAAACTTGA |
| *SlACO2* | [1-Aminocyclopropane-1-carboxylic Acid Oxidase](http://fjour.blyun.com/views/specific/3004/FJourDetail.jsp?dxNumber=165198978170&d=51DC9D17B8D210C15454F6F09E223FBC&s=ACO1)2 | CGTAGACCCTGAAACTGGTG | | GTGTAGCCATCCTTCCTTGTC |
| *SlACO3* | [1-Aminocyclopropane-1-carboxylic Acid Oxidase](http://fjour.blyun.com/views/specific/3004/FJourDetail.jsp?dxNumber=165198978170&d=51DC9D17B8D210C15454F6F09E223FBC&s=ACO1)3 | ACGAATCCCTTCCCAGAAA | | TCTTTGTCTATGGTCCTCTCAGTC |
| *SlACS1* | 1-Aminocyclopropane-1-carboxylic cid Synthase 1 | CATACCCCGACTACTACTTTCGT | | CGCACATACTGGGATTCTCTT |
| *SlACS2* | 1-Aminocyclopropane-1-carboxylic cid Synthase 2 | AGCGAAAGCCATTGAAAAGT | | CATTTGGTGATTGTGTGAAGAG |
| *SlE4* | Enhancer 4 | CTTGGCTTGGGATTAGAAGAAG | | TGAGGGCATTTTGGGTAGTAGT |
| *SlE8* | Enhancer 8 | CTTCTGTGGTGCTGTTTGATG | | AGGGTGGTGCTTCAGTTTATGT |
| *SlCHS* | Chalcone synthase | TCAACTCGTCAATGGCAGAC | | GAATGCTCGTATCAACCAGAGA |
| *SlF3H* | Flavanone 3-hydroxylase | GCAAGTAGAGTCGCACAGTTTT | | TCAGTATTAGGCTTCCCACCA |
| *SlANS* | Anthocyanidin synthase | GCTCTGTTTTGCCTGGACTT | | GCGTGGCTTAGATAGTTGGAAT |
| *SlPSY* | Phytoene synthase | ATGGAAAGCCTACTTGTGCTG | | CTGGTGTAATGTTGTTGGTCGT |
| *SlSPS1* | Sucrose phosphate synthase | AGGACGGAGTTGGATTGC | | TGAGCGTGAGCGTGAAG |
| *SlSS1* | Sucrose synthase | GTATCGTCCCCGTCTCATTC | | AGTAGGAGTGCCCGTTGATT |
| *SlPG1* | Polygalacturonase | TCACTGCCACTAACTTCTGC | | TTATGCCTCCTGCTCTCCT |
| *SlPME1* | Pectinesterase | TACAGCCAGGGGTATGGAAC | | GTTAGGGAGGGCATTGTTTG |
| *SlXYL1* | B-xylosidase1 | CTTCTTACTCTGACCCCATTGTTT | | TGTGCTTAGTGCTGCTGTGTT |
| *SlEXP1* | Expansin1 | CACTGACTCTCCCCTACCTACAAT | | ATACACTTCATCCCCCACCTTA |
| *SlCEL2* | Cellulose 2 | CCCTGATTCTGACCTTTACTGG | | GATGATGAAGTCGGTGTGGTT |
| *SlCEL4* | Cellulose 4 | CGTAGATTGGAGTTATGGAGAGC | | CGAATGATGTAAGAACCACTGC |
| *SlRIN* | Ripening inhibitor | GGAACCCAAACTTCATCAGA | | TTGTCCCAAATCCTCACCTA |
| *SlCNR* | Colorless nonripening | ACTGAAGCTGAAAAGGAAGAGGA | | TAATTTTACGGCGGCGCTCA |
| *SlHB1* | HD-Zip homeobox protein | GGGGAAAGTGGTTGGAGGAG | | TCCTTGTTCATGGTGCTGCT |
| *SlACTIN* | Actin protein | GAGTGTTTGTGTTTGGGTTTTG | | CGATGGTCCTTTCCAGTGA |
| *MdACO1* | [1-Aminocyclopropane-1-carboxylic Acid Oxidase](http://fjour.blyun.com/views/specific/3004/FJourDetail.jsp?dxNumber=165198978170&d=51DC9D17B8D210C15454F6F09E223FBC&s=ACO1)1 | TCAAGGATGGTGAATGGGTG | | AATGAGTCGTTGCCTGGGTT |
| *MdACO2* | [1-Aminocyclopropane-1-carboxylic Acid Oxidase](http://fjour.blyun.com/views/specific/3004/FJourDetail.jsp?dxNumber=165198978170&d=51DC9D17B8D210C15454F6F09E223FBC&s=ACO1)2 | TCGGACGGAACCAGAATG | | CTCCTTGGCTTGGAATTTGA |
| *MdACO3* | [1-Aminocyclopropane-1-carboxylic Acid Oxidase](http://fjour.blyun.com/views/specific/3004/FJourDetail.jsp?dxNumber=165198978170&d=51DC9D17B8D210C15454F6F09E223FBC&s=ACO1)3 | AGCAAAGGTTCAAGGAGCTG | | TCCAATTTCAATGCAAACTCC |
| *MdACS1* | 1-Aminocyclopropane-1-carboxylic cid Synthase 1 | CTGGACTTGGCAATGCAGA | | CGGGAAACCCACTTTGTGA |
| *MdACS3* | 1-Aminocyclopropane-1-carboxylic cid Synthase3 | TGAGTCAAGCAACCCATCTG | | AATTTGCCATTGCCTTTCTG |
| *MdACS5* | 1-Aminocyclopropane-1-carboxylic cid Synthase5 | GGATTGTTATGAGCGGAGGA | | TGCCTTCTGAGCCTTCTCAT |
| *MdACS6* | 1-Aminocyclopropane-1-carboxylic cid Synthase 6 | TGGATTGTCCGAGAACAGGC | | TGAACGGGAATCAGCTCCAC |
| *MdETR2* | Ethylene response2 | GCACAATAGCAAGGTCTGAGC | | CTCGGAGACAACTTAACCAAAG |
| *MdETR5* | Ethylene response5 | GAGTGCTCTTACTGCCTCCG | | CGCACTTGCTGTGATGGCT |
| *MdERS1* | [Ethylene response sensor 1](http://fjour.blyun.com/views/specific/3004/FJourDetail.jsp?dxNumber=165186737649&d=D0E68003506DD66BC5CB1929F3A59F73&s=ERS1) | GTTGCATTCATCGTCCAACTA | | CGAAACAATCCCTAAAGGCG |
| *MdCTR1* | Constitutive triple response 1 | ACAAGATTTTCATGCCGAAC | | TATGGACAAGTTTGGAGGCT |
| *MdERF1* | Ethylene response factor1 | ACAAAGAGGCGGTGCATTGTTG | | CACCACCGCCGTGATATAATGGA |
| *MdERF2* | Ethylene response factor2 | TATGCTGGCAATTGGCGAGC | | ATGACCAATCCCGCACTCAC |
| *MdERF3* | Ethylene response factor3 | TGGAGCTGACTCGGACTACA | | CCGACTTCTGAACCGGTTGA |
| *MdEIN2A* | Ethylene insensitive2a | GAGCCGCAGTACCATTCTTC | | CCTGAAGCCGGTTGAGAAC |
| *MdBG* | Beta glucohexaose glycosidase | AGATACACTGGGTGGCTTGACG | | GGTGCCTGGAGACCTACATCG |
| *MdCEL4* | Carboxyl ester lipase 4 | GGTGGACCTGATGCCTATGAC | | GGCTGGGATGTAACAACTGGA |
| *MdPG1* | Polygalacturonase 1 | TAAGTATCGGAAGTCTCGGAAGG | | CCGCAGTTGAAGTTGTCCCTAT |
| *MdEXP1* | Expansin 1 | GGTGCTTGTGGATATGGAAACC | | AGAACATGGGCATGGCGAAG |
| *MdXYL1* | Alpha-xylosidase 1 | ACACTACCTTCCGCCACTCACTA | | GAATCCCAAACTGGTCTACAACG |
| *MdPME1* | Pectin methylate esterase 1 | GTTGGACCACATTTCGCTCTG | | CTCGGTAGAACTGGCGCATAGA |
| *MdEXP 2* | Expansin 2 | CTTGCTACGAAATGCGATGTGA | | AAGTAGGAGTGCCCGTTGATTG |
| *MdSS1* | Sucrose synthase | CAGTGTACTATTGCGCACGC | | TGACGAGGTTTGCCAGATCC |
| *MdSPS1* | Sucrose phosphate synthase 1 | GGGGATGGTGAAGGGGAAAG | | TGCTGATCATGGGGGTCAAC |
| *MdMYC2* | Myelocytomatosis protein | CAAGTGTTTGGGCTGCAGAC | | GGCTCGGGTTCTCGGATAAG |
| *MdMYB10* | Myb domain protein 10 | TTGGAACACTCGATTGCGGA | | AATTGGCGCATGATCTTGGC |
| *MdCHS* | Chalcone synthase | GGAGACAACTGGAGAAGGACTG | | CGACATTGATACTGGTGTCTTCA |
| *MdDFR* | Dihydroflavonol 4-reductase | GATAGGGTTTGAGTTCAAGTA | | TCTCCTCAGCAGCCTCAGTTTTC |
| *MdUFGT* | Flavonoid 3-O-glucosyltransferase | CCACCGCCCTTCCAAACACTCT | | CACCCTTATGTTACGCGGCATGT |
| *MdVHA* | Vacuole H^+^-ATPase | CTACCATGGCTTGCCAGAAT | | ATCACCAGGATCAGCCAAAC |
| *MdSBE* | Starch branching enzyme | GGCCATCCCTGACAAATGGA | | CATAACTCCACCCGTTGCCT |
| *MdSSS* | Starch synthase | ATAACCGAGGCTCAGGTCCT | | GTTCTTTCAGCCTTGGCACG |
| *MdGBSS* | Granules bound Starch synthase | TGTCCCTCTGGCCCATATGA | | CCCTCAATCCCAAGTTCGCT |
| *MdAGPP* | AGP pyrophosphorylase | GAGTGGCGCCGATATTACCA | | ATTTGCTGTCGGAAAACGCC |
| *MdSAMS* | SAM synthetase | AATCCGTGAACGAGGGTCAC | | GGGGTTTCGTCAGTGGCATA |
| *MdACTIN* | Actin protein | AGGTCCATCCATTGTCCACAG | | TGCCAACCAAACTGACTTCAC |

**Supplementary Table S4**: Primers used for BiFC, subcellular localization, and Co-IP assays.

| **Gene** | **Forward primer (5’-3’)** | **Reverse primer (5’-3’)** |
| --- | --- | --- |
| pCambia1300:MdSAM-YFPN | GGCGCGCCATGGAGACTTTCCTATTCACATCTGAATC | ACTAGTAGACTGAGGCTTCTCCCACTTGAGG |
| pMDC83: MdFERL6 | TTAATTAAATGAAGTGTTTCTTTTTCTATATTTGGTTC | GGCGCGCCAACGTCCCTTTGGGTTCATGATTTGTGAG |
| pCambia1300:MdFERL1-YFPC | GGCGCGCCATGATTATGAACACTGCTGGGTTTATAG | ACTAGTACGTCCTTTAGGGTTCATGATTTGTGAGAACACGGCACT |
| pCambia1300:MdFERL6- YFPC | GGCGCGCCATGAATCCCAATCTTGCTCATCTGTTC | ACTAGTCCTTCCATTGGGATCGTTGATCTCAG |
| pCambia1300:MdETR2-YFPN | GGTACCATGTTAAAGGCATTGGCATCTTCG | GGATCCTGTCATCCCTTTGTTTGCCTGC |
| pCambia1300:MdETR5-YFPN | GGTACCATGTTCAGAGCATTAGCAATTGGA | GGATCCATCTTTTACCGACATCTCCATCAC |
| pCambia1300:MdACS3-YFPN | GGTACCATGGCTATAGATATTGAGCAGCGGCAG | GGATCCGCAAGCCCGCTCTCTCTTTTCC |
| pCambia1300:MdACS5-YFPN | GGTACCATGGGGTTTACTTTGAGCAACCAACAG | GGATCCAGTGGCTCGAACAAGAGGCGATTG |
| pCambia1300:Myc-MdSAM-YFPN (CoIP) | GGCGCGCCATGGAGCAGAAACTCATCTCTGAAGAGGATCTGGAGACTTTCCTATTCACATCTGAATC | ACTAGTAGACTGAGGCTTCTCCCACTTGAGG |
| pMDC83:His-MdFERL6-GFP (CoIP) | TTAATTAAATGAAGTGTTTCTTTTTCTATATTTGGTTC | GGCGCGCCAACGTCCCTTTGGGTTCATGATTTGTGAG |

**Supplementary Table S5**: RT-PCR primers used for detecting *MdFERLs* expression.

| **Gene** | **Genbank**  **accession number** | **MDP**  **accession number** | **Forward primer (5’-3’)** | **Reverse primer (5’-3’)** | |
| --- | --- | --- | --- | --- | --- |
| *MdFERL1* | KY435591 | MDP0000445374 | AATTGCCTTAGCCCTCGTCC | CACGCAGGGTTCCATAAGCC |  |
| *MdFERL2* | KY435586 | MDP0000247000 | TGATTACATGGCTTACGGAA | TCTTGAAGCACTCTGGCGTTATC |  |
| *MdFERL3* | KY435598 | MDP0000420638 | CGCCGAGTTCGACCGATCTA | ATCTGCTCCGCCGTCTGATT |  |
| *MdFERL4* | KY435587 | MDP0000147000 | TCCGTAGGCAGCATAAACC | ATGAAGATTTGAAACAGCCGGT |  |
| *MdFERL5* | KY435596 | MDP0000234336 | ATCTTCATCGCCAATCAGAC | GTAAAGAAGAGCCGCGAGTC |  |
| *MdFERL6* | KY435598 | MDP0000465341 | CTCTCTCGCTCCGAATTTAC | TTGCGGCAGAACACTAAAGT |  |
| *MdFERL7* | KY435592 | MDP0000870778 | CCACCGCAATACAATAGCAG | GTTTCAGCCGTTTGATAGCA |  |
| *MdFERL8* | KY435597 | MDP0000450994 | TCCACGACTTCAACGCTTCA | ATTGAGCGGCCTGATTGGTA |  |
| *MdFERL9* | KY435595 | MDP0000161440 | CCATACGCAACAGCTACAAT | CCATACGCAACAGCTAC |  |
| *MdFERL10* | KY435589 | MDP0000413728 | CCTTATTCCGGCGAAGTGTCT | TGCGGTACAGACCAGTATCG |  |
| *MdFERL11* | KY435594 | MDP0000297735 | TCCACGACTTCAACGCTTCA | CCCAATTGCGGTACATACC |  |
| *MdFERL12* | KY435594 | MDP0000493959 | CCAATCAAACGGCGGAAACA | ATCGGACGGCAGAGTTGAGC |  |
| *MdFERL13* | KY435597 | MDP0000427444 | GCCCAGGAGTTCAAGACGGA | TGCAACTCATCGCGACGTCA |  |
| *MdFERL14* | KY435596 | MDP0000816012 | CAACAAGAAAGTATGCACC | GCCAACCATTGACACAACG |  |
| *MdFERL15* | KY435602 | MDP0000721825 | AACCCTGCAATCCCGAAGAA | CAGCTCGATTTCCGTCTTGA |  |
| *MdFERL16* | KY435603 | MDP0000451269 | CCCAAAGTTCATCCGCTTGT | TGCTATTGTATTGCGGTGGT |  |
| *MdFERL17* | KY435600 | MDP0000310638 | TCCGCTCGGTTTCTTATGGC | ATTCCGCGCTAATCGACAGG |  |
| *MdACTIN* | [XM_008393049.2](https://www.ncbi.nlm.nih.gov/nucleotide/1039843842?report=genbank&log$=nuclalign&blast_rank=25&RID=NGTH2GG9014) | MDP0000774288 | TGGTTGGTATGGGTCAGAAG | CAGCAAGGTCCAGACGAAG |  |

**Supplementary Figure**


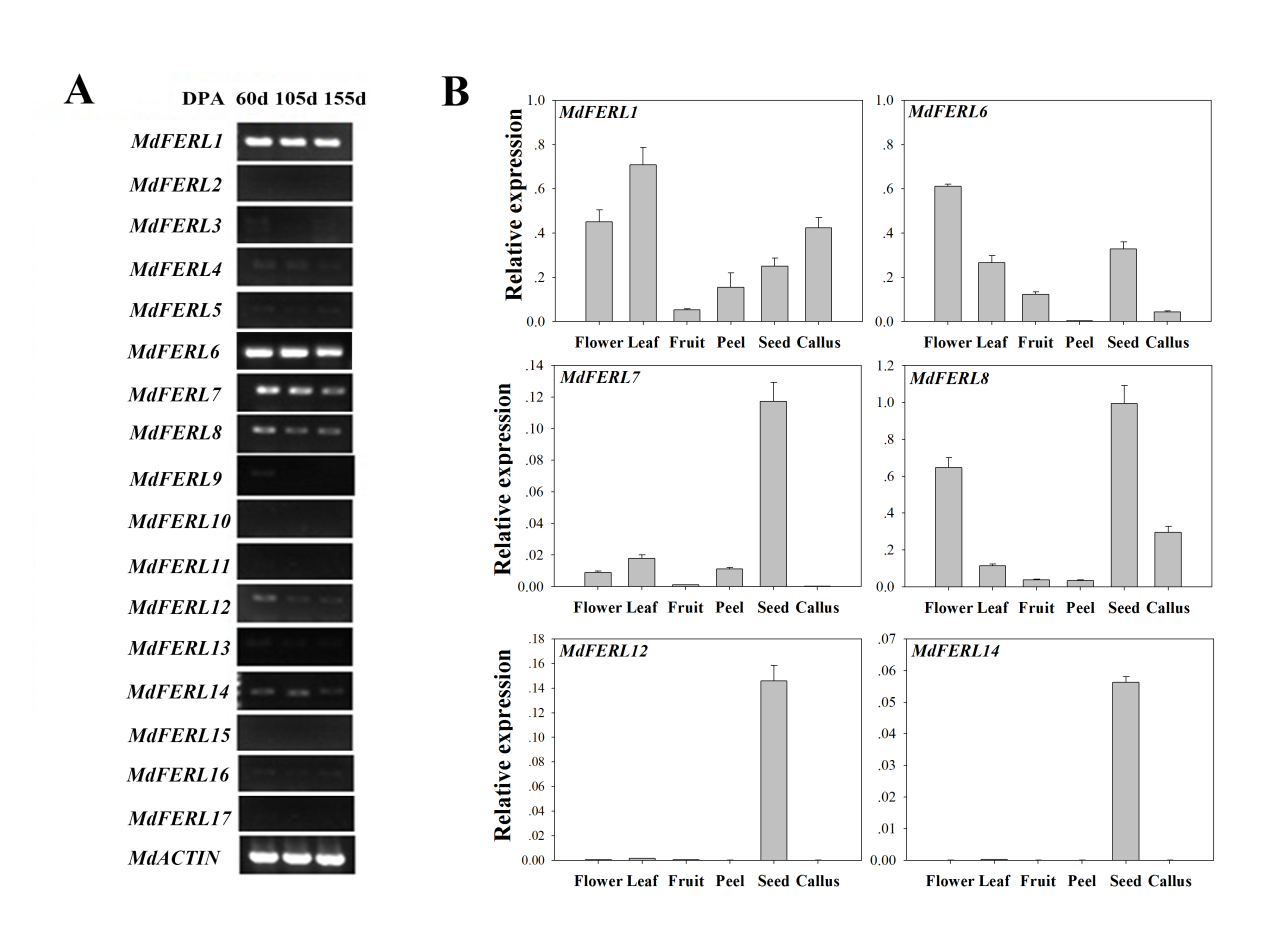


**Figure S1 | Expression analysis of *MdFERLs*.** (A) Reverse transcription polymerase chain reaction (RT-PCR) analysis of *MdFERLs* at the indicated developmental stages. DPA, days post-anthesis. (B) Tissue-specific expression of *MdFERLs* in apple.

**
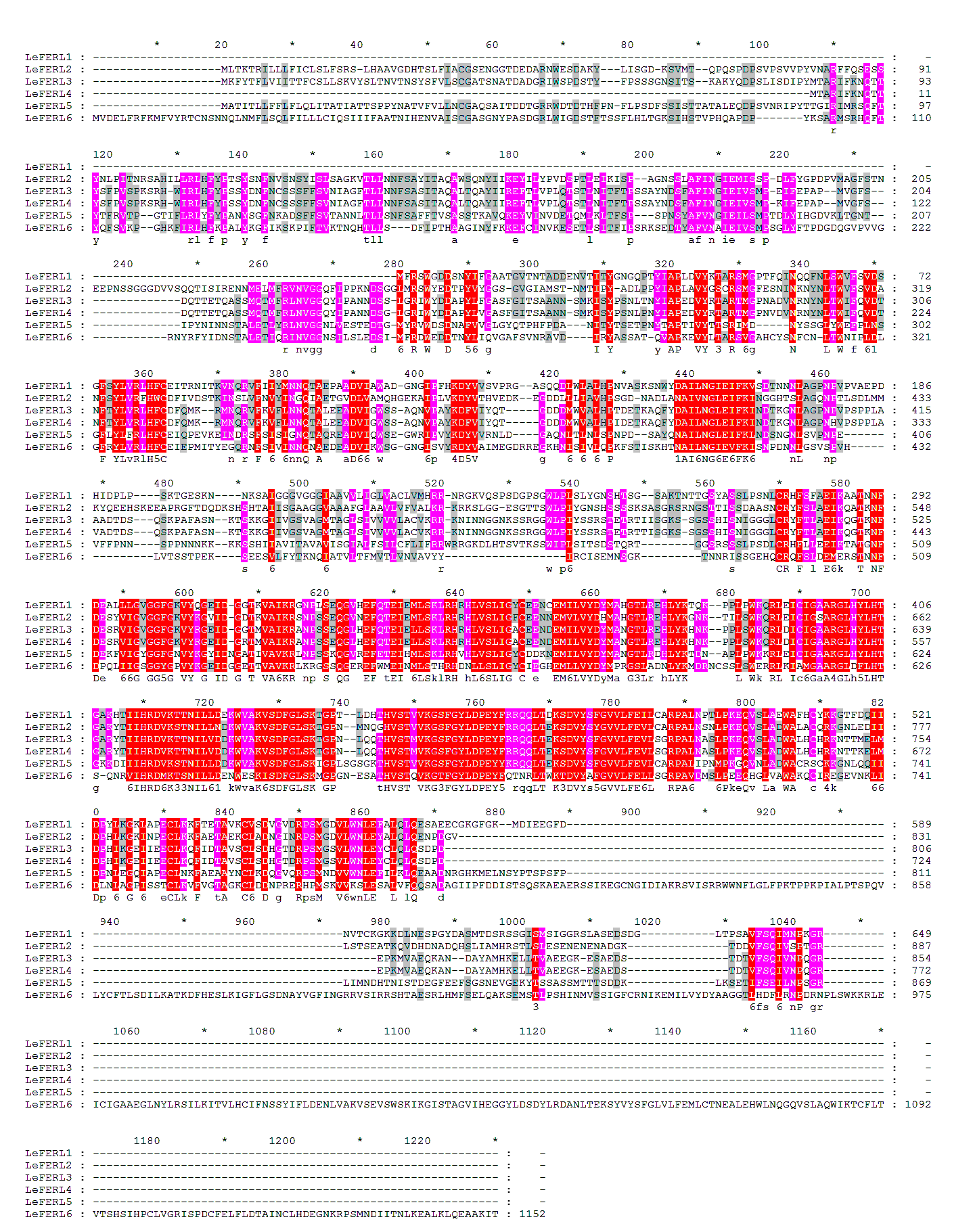
**

**Figure S2 | Sequence alignments of the deduced SlFERL amino acid sequences.** Sequences were aligned using ClustalX 2.0.12 with default settings. The alignments were edited and marked using GeneDoc. Black and light-gray shading indicate identical and similar amino acid residues, respectively.

**
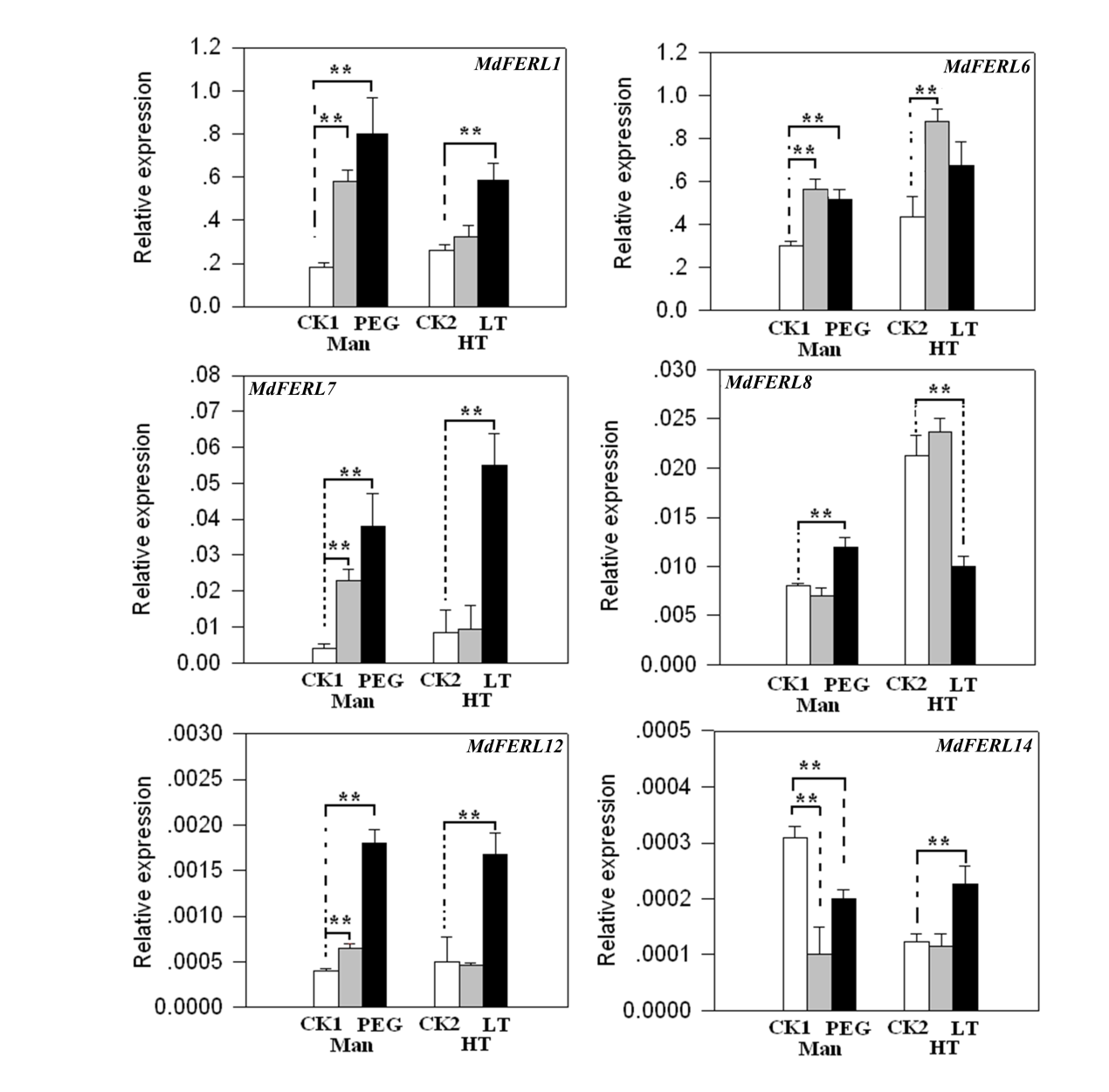
**

**Figure S3 | RT-qPCR analysis of MdFERL expression in response to mannitol (Man), polyethylene glycol (PEG), low temperature (LT), and high temperature (HT) treatments in fruits at 105 days post-anthesis (DPA).** RT-qPCR was conducted using *MdACTIN* as an internal control. Values are means + SD of three biological replicates. **, P < 0.01 (Student’s *t*-test), when compared with control values (CK1: control of Man and PEG; or CK2: control of LT and HT).
